# Proteomic Analysis of Arabidopsis *pld*α*1* Mutants Revealed an Important Role of Phospholipase D Alpha 1 in Chloroplast Biogenesis

**DOI:** 10.3389/fpls.2019.00089

**Published:** 2019-02-18

**Authors:** Tomáš Takáč, Tibor Pechan, Olga Šamajová, Jozef Šamaj

**Affiliations:** ^1^Faculty of Science, Centre of the Region Haná for Biotechnological and Agricultural Research, Palacký University, Olomouc, Czechia; ^2^Institute for Genomics, Biocomputing and Biotechnology, Mississippi Agricultural and Forestry Experiment Station, Mississippi State University, Starkville, MS, United States

**Keywords:** phospholipase D alpha 1, proteomics, Arabidopsis, chloroplast biogenesis, translation, chloroplast protein import

## Abstract

Phospholipase D alpha 1 (PLDα1) is a phospholipid hydrolyzing enzyme playing multiple regulatory roles in stress responses of plants. Its signaling activity is mediated by phosphatidic acid (PA) production, capacity to bind, and modulate G-protein complexes or by interaction with other proteins. This work presents a quantitative proteomic analysis of two T-DNA insertion *pld*α*1* mutants of *Arabidopsis thaliana*. Remarkably, *PLD*α*1* knockouts caused differential regulation of many proteins forming protein complexes, while PLDα1 might be required for their stability. Almost one third of differentially abundant proteins (DAPs) in *pld*α*1* mutants are implicated in metabolism and RNA binding. Latter functional class comprises proteins involved in translation, RNA editing, processing, stability, and decay. Many of these proteins, including those regulating chloroplast protein import and protein folding, share common functions in chloroplast biogenesis and leaf variegation. Consistently, *pld*α*1* mutants showed altered level of TIC40 (a major regulator of protein import into chloroplast), differential accumulation of photosynthetic protein complexes and changed chloroplast sizes as revealed by immunoblotting, blue-native electrophoresis, and microscopic analyses, respectively. Our proteomic analysis also revealed that genetic depletion of PLDα1 also affected proteins involved in cell wall architecture, redox homeostasis, and abscisic acid signaling. Taking together, PLDα1 appears as a protein integrating cytosolic and plastidic protein translations, plastid protein degradation, and protein import into chloroplast in order to regulate chloroplast biogenesis in Arabidopsis.

## Introduction

Phospholipase D alpha 1 (PLDα1) belongs to a family of phospholipases involved in many biotic and abiotic stress responses of plants (Li et al., 2009; Zhao, 2015). It is a phospholipid degrading enzyme, which in the presence of alcohol can catalyze also transphosphatidylation reactions. Most plant PLDs, including members of the α, β, γ, δ, ε, and κ (also known as θ) subfamilies, contain a Ca^2+^/phospholipid-binding C2 domain near the N-terminus (Hong et al., 2016). Members of PLDα subfamily require Ca^2+^ in millimolar range, thus differing from other PLDs. Unlike other PLDs, PLDα1 does not contain plekstrin homology (PH), phox homology (PX), or phosphatidylinositol 4,5-bisphosphate (PIP2)-binding domain. Members of PLDα subfamily do not contain actin binding region which is present in members of PLDβ subfamily. Phospholipases utilize a wide range of phospholipid substrates, while PLDα1 preferentially degrades phosphatidylcholine, and in smaller extent phosphodiethanolamine.

PLDα1 has been detected in cytoplasm of non-dividing cells, while it can be enriched in mitotic spindles and phragmoplasts of meristematic cells (Novák et al., 2018). It was also found by subcellular proteomic studies in isolated chloroplasts (Kleffmann et al., 2004; Zybailov et al., 2008), endosomes (Heard et al., 2015), and plasma membrane (Elmore et al., 2012). Recently, detailed observation of *pld*α*1* mutants carrying *proPLD::PLD*α*1:YFP* construct showed that PLDα1 is localized together with microtubules and clathrin in the vicinity of plasma membrane, and it is enriched in this location after salt stress (Novák et al., 2018). From developmental point of view, *PLD*α*1* is strongly expressed in the root cap, rhizodermis (preferentially in trichoblasts), and it accumulates in the tips of growing root hairs and leaf trichomes (Novák et al., 2018).

Function of PLDα1 is modulated by protein-protein interactions. For example, it interacts with components of G-protein complex. These combinatorial interactions affect developmental processes and abscisic acid (ABA) signaling pathway. PLDα1 primarily acts as a GTPase-activating protein (GAP) for Guanine nucleotide-binding protein alpha-1 subunit (GPA1), and the role of RGS1 (Regulator of G-protein signaling 1) is likely to inhibit the GAP activity of PLDα1 (Gookin and Assmann, 2014; Pandey, 2016; Roy Choudhury and Pandey, 2016). It was later shown that PLDα1 may also, via phosphatidic acid (PA) binding mechanism, affect RGS1 (Roy Choudhury and Pandey, 2017). PLDα1 is likely sensitive to redox regulation, since important redox signaling molecules such as hydrogen sulfide and nitric oxide affect PLDα1 mediated PA production (DistéFano et al., 2007; Scuffi et al., 2018).

PA, as a product of PLD activity, has a multiple signaling roles in plants (Testerink and Munnik, 2011; Hou et al., 2016). However, PA is also produced by PLCs (Singh et al., 2015) and diacylglycerol kinases (Arisz et al., 2009). The glycerol phosphate pathway located in endoplasmic reticulum, mitochondria, and chloroplast serves as a PA pool devoted for glycerophospholipid and triacylglycerol synthesis (Athenstaedt and Daum, 1999; Testerink and Munnik, 2011). Generally, PLDα1 deficiency causes rearrangements in lipid composition (Devaiah et al., 2006) and lowers PA level (Sang et al., 2001; Zhang et al., 2009b; Uraji et al., 2012).

Concerning physiological functions, PLDα1 is involved in stomatal closure, ABA (Zhang et al., 2004, 2009b; Uraji et al., 2012; Jiang et al., 2014), ethylene (Testerink et al., 2007), and salicylic acid signaling (Janda et al., 2015), response to salinity (Bargmann et al., 2009; Yu et al., 2010; Novák et al., 2018), cold and freezing stress (Rajashekar et al., 2006; Huo et al., 2016), and production of superoxide (Sang et al., 2001; Zhang et al., 2009b).

These PLDα1 functions are most often assigned to the ability of proteins to bind to PA. So far, several proteins interacting with PA have been identified to have roles in abiotic stress responses of plants. These include ABI1 phosphatase 2C (Zhang et al., 2004), mitogen activated protein kinase 6 (Yu et al., 2010), constitutive triple response 1 (Testerink et al., 2007), NADPH oxidase (Zhang et al., 2009b), and sphingosine kinases (Guo et al., 2011).

One very important role of PLDα1 and PA is their ability to modulate actin and microtubule cytoskeletons (Pleskot et al., 2013). This regulation is mediated via targeting of actin- or microtubule-binding proteins by PA. Thus, microtubule associated protein 65-1 (MAP65-1) and actin capping protein 1 were identified as PA binding proteins (Huang, 2006; Zhang et al., 2012). Interestingly, interaction between PA and MAP65-1 contributes to better salt stress tolerance due to the microtubule stabilization (Zhang et al., 2012).

Here, we present a comparative proteomic analysis of two T-DNA insertion *pld*α*1* mutants of *Arabidopsis thaliana* in order to uncover novel regulatory roles of PLDα1.

## Methods

### Plant Material

Seeds of *Arabidopsis thaliana*, ecotype Col-0 and two T-DNA insertion lines *pld*α*1-1* (SALK_067533) and *pld*α*1-2* (SALK_053785) described recently (Novák et al., 2018) were obtained from Nottingham Arabidopsis Stock Center (Nottingham, UK). Following ethanol-sterilization, they were cultivated on solid 1/2 MS media at 21°C under 16/8 light/dark illumination conditions. Above ground parts of 14 days old seedlings were harvested for proteomic analysis, immunoblotting, chlorophyll content measurements and chloroplast analyses.

### Protein Extraction and Trypsin Digestion

Four replicates for each of the three biological samples (leaves of Col-0, *pld*α*1-1, and pld*α*1-2*) were subjected to proteomics analyses. Each replicate consisted of 30 seedlings. To limit the effects of variations between individual plants, the specimens were pooled.

Plant material was homogenized using liquid nitrogen, mortar and pestle, and subjected to phenol protein extraction followed by methanol precipitation as described in Takáč et al. (2011). Protein pellets were dissolved in 6 M urea in 50 mM Tris-HCl (pH 7.4) and the protein concentration was measured by Bradford assay (Bradford, 1976). In total, 100 μg (in 50 μl volume) of proteins was used for trypsin digestion per sample. Prior to trypsin digestion, proteins were reduced by addition of 50 mM dithiothreitol followed by alkylation with 50 mM iodoacetamide. Both reactions were performed at room temperature (RT) for 1 h. After lowering the urea concentrations to 1 M by 50 mM Tris-HCl (pH 7.4), proteins were digested at 37°C overnight using sequencing grade trypsin (Promega, Madison WI, USA) in amount of 1 μg per 50 μg of proteins. The digestion was stopped by addition of acetic acid. Tryptic digests were subsequently cleaned on C18 cartridges (Bond Elut C18; Agilent Technologies, Santa Clara, CA) according to manufacturer's instructions. Finally, cleaned peptides eluted by 95% (v/v) acetonitrile were dried using vacuum concentrator and stored under −80°C until analysis.

### Liquid Chromatography, Mass Spectrometry, Protein Identification, and Relative Quantitative Analysis

LC-MS/MS and protein identification was performed as published previously (Takáč et al., 2017). Briefly, two micrograms of protein tryptic digest were loaded on reversed phase Acclaim PepMap C18 column (Thermo Fisher Scientific, Waltham, MA, USA). A constant flow (0.3 μl.min^−1^), 170 min long non-linear gradient of acetonitrile in 0.1% (v/v) formic acid (2–55% for 125 min, 95% for 15 min, 2% for 30 min) was used to separate and elute peptides. The mass spectra were collected in the data dependent acquisition mode in 18 scan events: one MS scan (m/z range: 300–1,700) followed by 17 MS/MS scans for the 17 most intense ions detected in MS scan (with dynamic exclusion being applied). The method and raw spectral files were created and generated, respectively, by Xcalibur 2.1 (Thermo Fisher Scientific). The files were analyzed using the SEQUEST algorithm of the Proteome Discoverer 1.1.0 software (Thermo Fisher Scientific). Variable modifications were set as follows: cysteine carbamidomethylation (+57.021), methionine oxidation (+15.995), methionine dioxidation (+31.990). The spectral data were matched against target and decoy databases for more stringent approach to estimate false discovery rates (FDR), compared to single search of concatenated database. The UNIPROT (www.uniprot.org) Arabidopsis genus taxonomy Reviewed protein database (17,586 entries as of September 2017) served as the target database, while its reversed copy (created automatically by the software) served as a decoy database. The search results were filtered by FDR <1%. Identified proteins were grouped by default parameters of the software, defining the group as proteins strictly necessary to explain presence of identified peptides. A representative/master protein of the group is the protein with highest score, spectral count and number of matched peptides. If those parameters are equal, the protein with longest sequence is designated as a master protein. Proteins listed in the Supplementary Material are master proteins. All proteins, their accession numbers, respective peptides, and annotated spectra are included in “msf” files (see below how to view them). If the peptide can be attributed to more than one protein, it is indicated by multiple protein accession numbers allocated to the given peptide. This is also shown in Supplementary Material.

The relative quantitative analysis was done using the ProteoIQ 2.1 (NuSep) software as published previously (Takáč et al., 2016). It was based on sums of precursor ion intensities (PII) of filtered peptides attributed to given proteins. Summed intensities pertinent to proteins in individual replicates were normalized by factors that were calculated to equalize total intensity of all master proteins across all biological samples and replicates. Normalized average protein intensities were used to calculate fold changes when comparing biological samples. All data points were considered. The ANOVA *p* ≤ 0.05 was used to filter statistically significant results. Proteins with fold change higher than 1.5 were considered as differentially abundant. Proteins identified by 1 peptide in one of the two mutants (the same protein being identified with high stringency in the second mutant) were also considered as commonly differentially abundant in both mutants.

### Bioinformatic Analysis

Gene ontology (GO) annotation analysis of differentially abundant proteins (DAPs) was performed by Blast2Go software (Conesa and Götz, 2008). Blast was performed against *Arabidopsis thaliana* NCBI database allowing 1 BLAST Hit. The annotation was carried out by using these parameters: *E*-Value Hit filter: 1.0E^−6^; Annotation cut off: 30; GO weight: 5. GO SLIM function was used to reduce the GO annotation number. Blast2Go platform was also used to perform classification of metabolic functions according to KEGG (Kyoto Encyclopedia of Genes and Genomes) pathways. STRING (Szklarczyk et al., 2015) database was used for analysis of protein interaction network applying minimum required interaction score 0.55, relevant for high confidence prediction. Only experimentally approved interactions, including interactions between heterologous proteins, were considered. The prediction of presence of chloroplast transit peptide in the differential proteomes of the mutants was performed using ChloroP 1.1 server (Emanuelsson et al., 1999).

### Cycloheximide Treatment

Seeds of wild type and *pld*α*1-1* and *pld*α*1-2* mutants were placed on solid 1/2 MS media supplemented with 0.4 μM cycloheximide (Sigma-Aldrich, Heidelberg, Germany) dissolved in 96% (v/v) ethanol and incubated for 1 day at 4°C in the dark for stratification. As controls, seeds were placed on media supplemented with 0.005% (v/v) ethanol. Plants were grown vertically, and illumination of their roots was prevented by opaque black foil. Fresh weight of individual plants (*n* = 15 in each of the three biological replicates) was measured after 16 days of incubation. One-way Anova analysis was used for statistical evaluation of differences between wild type and mutants.

### Immunoblotting

Urea extract aliquots (from proteomic analysis) were enriched by 4 times concentrated Laemmli buffer to reach final concentration of 62.5 mM Tris-HCl (pH 6.8), 2% (w/v) SDS, 10% (v/v) glycerol, 300 mM 2-mercaptoethanol) and used for immunoblotting. Extracts (20 μg of proteins per sample) were separated on 10% SDS-PAGE gels and proteins were transferred to nitrocellulose membranes by semi-dry transfer using Trans Blot Turbo apparatus (BioRad). The protein transfer was validated by reversible staining of proteins on the membrane using Ponceau S. After destaining by three washes in Tris buffered saline (pH 7.4) with 0.1% (v/v) tween 20 (TBST), membranes were incubated in 15% (v/v) Western blocking reagent (Roche, Basel, Switzerland) overnight at 4°C, followed by overnight incubation (at 4°C) with anti-Tic40 primary antibody (PhytoAB, San Francisco, CA, USA) diluted using TBST buffer containing 4% (v/v) Western blocking reagent. Membranes were then thoroughly washed five times in TBST using fast agitation and incubated in horseradish peroxidase-conjugated (F(ab')_2_ goat anti-rabbit IgG (H+L) secondary antibody (Thermo Fisher Scientific) diluted to 1:5000 in TBST with 4% (v/v) Western blocking reagent at RT for 1.5 h. Following thorough five times repeated washing in TBST, the signal was developed using Clarity^TM^ ECL Western blotting substrate (BioRad, Hercules, CA, USA) and recorded with ChemiDoc^TM^ documentation system (BioRad). Immunoblotting was carried out in three biological replicates. Chemiluminescent signal was quantified using ImageLab software (BioRad).

### Chlorophyll Content Measurement

Chlorophyll content was measured in ice-cold 100% (v/v) acetone extracts of above ground parts of Col-0, *pld*α*1-1*, and *pld*α*1-2* plants according to Lichtenthaler and Buschmann (2001). Experiments were performed in three biological replicates.

### Chloroplast Isolation

Chloroplasts were isolated as described by Flores-Pérez and Jarvis (2017) with modifications. Arabidopsis leaf material (4 g) was grinded in 10 ml of cooled chloroplast isolation buffer (CIB; 0.6 M sorbitol, 10 mM MgCl_2_, 10 mM EGTA, 10 mM EDTA, 20 mM NaHCO_3_, 40 mM HEPES-KOH, pH 8.0) using mortar and pestle. The mortar was flushed with additional 10 ml of CIB to collect the homogenate quantitatively. The homogenate was filtered through two layers of Miracloth (Merck Millipore, Burlington, MA, USA) into 50 ml centrifugation tube, and centrifuged at 1,000 *g* for 5 min at 4°C using fixed angle rotor. After gentle removal of the supernatant, the pellet was resuspended in the remaining volume of supernatant. The resuspended supernatant was overlaid onto pre-centrifuged (at 43,000 *g* for 30 min at 4°C in a fixed angle rotor, deceleration set to minimum) Percoll gradient (13 ml Percoll, 13 ml 2 × CIB, 5 mg reduced glutathione). It was centrifuged in a swinging bucket rotor at 4,000g at 4°C for 20 min (deceleration set to minimum). Intact chloroplasts appeared as a green sharp lower band. After removal of the upper layers, intact chloroplasts were collected by plastic Pasteur pipette into new 50 ml centrifugation tube. Suspension was diluted in 20 ml of HEPES-MgSO_4_-sorbitol (HMS) buffer (50 mM HEPES NaOH, pH 8.0, 3 mM MgSO_4_, 0.3 M sorbitol) and centrifuged at 1,000 *g* for 5 min at 4°C in a fixed angle rotor. Pelleted chloroplasts were resuspended in the remaining HMS buffer and stored at −80°C for further analyses. Ten μL of fresh suspension was used for confocal laser scanning microscopy observations.

### Visualization of Protein Complexes by Blue Native Electrophoresis

The protein content was measured by Bradford assay, and a volume containing an equal amount of proteins in each sample was used for chloroplast membrane solubilization as reported by Heinemeyer et al. (2004). First, a suspension containing 100 μg of proteins was centrifuged at 1,250 g at 4°C for 10 min. Pelleted chloroplasts were solubilized in 20 μl of sample buffer (30 mM HEPES, pH 7.4, 150 mM potassium acetate, 10% (v/v) glycerol, 2 mM PMSF and 1.5% (w/v) digitonin). After incubation on ice for 20 min, solubilized proteins were supplemented with 5 μl of 750 mM aminocaproic acid and 5% (w/v) coomassie briliant blue G250. The samples were loaded on 3–12% NativePAGE Bis-Tris Gels (Thermo Fisher Scientific) along with NativeMark Unstained Protein Standard (Thermo Fisher Scientific). Electrophoresis was run at constant 100 V in 50 mM Bis-Tris (pH 7.0) anode buffer and cathode buffer containing 50 mM Tricine, 15 mM Bis-Tris (pH 7.0), and 0.01% (w/v) Coomassie G250) at 4°C. When the protein front reached the middle of the gel, the cathode buffer was replaced with a similar but without Coomassie G250. Gel was stained using Bio-Safe Coomassie Stain and documented by Imagescanner III (GE Healthcare, Chicago, IL, USA). Experiments were performed in two biological replicates. Band intensities were evaluated using ImageJ software. One-way Anova analysis was used for statistical evaluation of differences between wild type and mutants.

### Observation of Chloroplast Autofluorescence by Confocal Laser Scanning Microscopy

Rosette leaves of similar sizes were cut from wild type Col-0, as well as *pld*α*1-1* and *pld*α*1-2* mutant plants and mounted in a drop of liquid ½ strength MS medium on microscope glass slides. Confocal laser scanning microscope (Zeiss LSM710) with laser 633 was used for imaging of chloroplasts, and autofluorescence of chlorophyll was observed at 650–720 nm. Three images from different locations per plant, and three plants for each plant line were randomly selected. Chloroplasts were quantitatively evaluated by measurements of their areas and diameter using Zen 2010 software (ZEISS, Oberkochen, Germany).

## Results

### Overview of Differential Proteomes in *pldα1* Mutants

**Figure 1:**
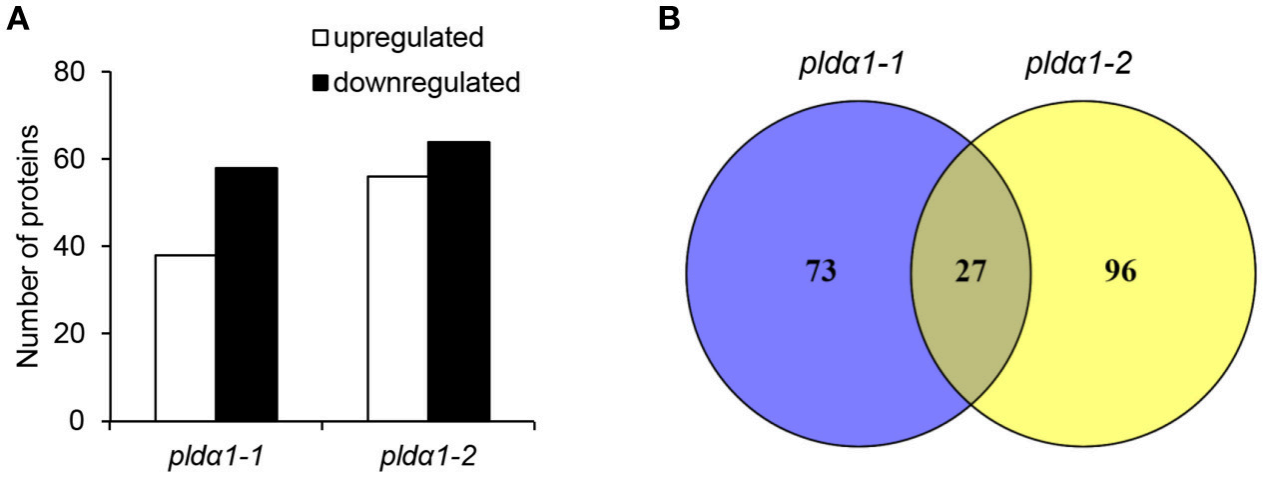
Overview of differential proteomes of *pld*α*1* mutants. **(A)** Numbers of proteins with increased and decreased abundance in *pld*α*1-1* and *pld*α*1-2* mutant above ground parts. **(B)** Venn diagrams showing differences in differential proteomes between the *pld*α*1-1* and *pld*α*1-2* mutants.

In this study, a comparative global proteomic analysis on above ground parts of two *pld*α*1* mutants as compared to the wild type plant was carried out. In total, 100 and 123 DAPs were found in *pld*α*1-1* and *pld*α*1-2*, respectively (Figure 1A). Twenty seven DAPs were commonly found in both mutants (Figure 1B). Information pertinent for protein identification in all samples is presented in Supplementary Data (Supplementary Material), and also deposited in PRIDE (see below).

The analyzed mutants were thoroughly tested by genotyping for the presence of *PLD*α*1* with reproducible negative results. We also performed a reverse transcript PCR showing no expression of *PLD*α*1* in the *pld*α*1-2* mutant (results not shown). Moreover, the current proteomic data showed the presence of PLDα1 protein only in the wild type (not in the mutants) and this was repeatedly occurring across the biological replicates (Table 1 and Table S1).

**Table 1:** List of proteins with significantly different abundances consistently found in *pld*α*1-1* and *pld*α*1-2* mutants as compared with wild type (Col-0).

**Sequence ID (UNIPROT)**	**Sequence ID (TAIR)**	**Sequence name**	***pldα1-1*/Col-0 ratio**	***pldα1-2*/Col-0 ratio**	***p*-Value *pldα1-1*/Col-0**	***p*-Value *pldα1-2*/Col-0**	**Total score^1^*pldα1-1*/*pldα1-2***	**Total peptides^2^*pldα1-1*/*pldα1-2***	**Total% seq coverage^3^*pldα1-1*/*pldα1-2***	**Total spectra^4^*pldα1-1*/*pldα1-2***
**CELL WALL**										
Q1JPL7	At1g11580	Pectinesterase/pectinesterase inhibitor 18	Unique in Col-0	0.59	n.a.	0.045	6.54/10.07	2/3	5.75/9.16	2/3
Q9FIE8	At5g59290	UDP-glucuronic acid decarboxylase 3	mutant unique	mutant unique	n.a.	n.a.	4.12/11.97	1/3	9.36/17.54	2/3
Q9SYM5	At1g78570	Trifunctional UDP-glucose 4,6-dehydratase/UDP-4-keto-6-deoxy-D-glucose 3,5-epimerase/UDP-4-keto-L-rhamnose-reductase RHM1	Unique in Col-0	Unique in Col-0	n.a.	n.a.	7.71/7.71	2/2	3.44/3.44	3/3
**TRANSLATION**										
F4IFC5	At2g04842	Threonine-tRNA ligase, chloroplastic/mitochondrial 2	mutant unique	mutant unique	n.a.	n.a.	10.45/7.71	3/2	7.08/5.08	3/2
O22793	At2g33430	Multiple organellar RNA editing factor 2, chloroplastic	0.30	0.46	0.01	0.007	7.90/7.90	2/2	16.44/16.44	8/8
**SECONDARY METABOLISM**										
Q9ZW84	At2g43100	3-isopropylmalate dehydratase small subunit 1	0.45	0.58	0	0.002	11.25/11.43	3/3	19.92/19.92	4/6
Q9M2E2	At3g61220	(+)-neomenthol dehydrogenase	1.95	2.60	0.022	0.026	16.50/22.76	4/6	26.69/30.74	11/20
**PROTEIN/PEPTIDE DEGRADATION**										
Q9FHW7	At5g42190	SKP1-like protein 1B	mutant unique	mutant unique	n.a.	n.a.	8.49/8.67	2/2	19.88/19.88	4/3
**PHOTOSYNTHETIC AND RESPIRATORY ELECTRON FLOW**										
P56759	AtCg00130	ATP synthase subunit b, chloroplastic	mutant unique	2.41	n.a.	0.004	7.04/12.00	2/3	18.48/28.80	3/9
Q9XFT3-2	At4g21280	Isoform 2 of Oxygen-evolving enhancer protein 3-1, chloroplastic	0.58	0.62	0.008	0.017	38.19/30.88	9/7	46.64/43.50	100/96
Q8H112	At4g22890	PGR5-like protein 1A, chloroplastic	6.28	6.77	0	0.001	11.71/15.29	3/4	14.81/14.81	9/9
**STRESS RESPONSE**										
Q38882	At3g15730	Phospholipase D alpha 1	Unique in Col-0	Unique in Col-0	n.a.	n.a.	12.06/12.06	3/3	8.15/8.15	8/8
Q9XEE2	At5g65020	Annexin D2	mutant unique	mutant unique	n.a.	n.a.	3.51/6.49	1/2	5.99/10.09	2/2
P22953	At5g02500	Probable mediator of RNA polymerase II transcription subunit 37e (HSP70-1)	0.73	0.65	0.031	0.003	137.76/127.79	32/29	57.91/57.91	226/209
Q9FK81	At5g22580	Stress-response A/B barrel domain-containing protein At5g22580	0.58	0.32	0.038	0.001	7.14/7.28	2/2	22.52/22.52	5/5
**PROTEIN FOLDING**										
Q9FF55	At5g60640	Protein disulfide isomerase-like 1-4	Unique in Col-0	3.84	n.a.	0.006	4.10/7.30	1/2	2.85/5.19	3/8
Q93ZM7	At3g13860	Chaperonin CPN60-like 2, mitochondrial	mutant unique	mutant unique	n.a.	n.a.	6.24/3.49	2/1	6.12/3.50	2/2
Q42406	At4g34870	Peptidyl-prolyl cis-trans isomerase CYP18-4	0.52	0.53	0.009	0.03	20.24/20.56	4/4	34.30/34.30	33/35
O22263	At2g47470	Protein disulfide-isomerase like 2-1	0.47	0.31	0.042	0.051	14.89/14.89	4/4	13.57/14	
**CHLOROPHYLL BIOSYNTHESIS**										
P16127	At4g18480	Magnesium-chelatase subunit ChlI-1, chloroplastic	0.53	0.52	0.046	0.021	41.47/44.51	10/11	36.56/39.62	50/54
**SIGNALING**										
O04153	At1g08450	Calreticulin-3	Unique in Col-0	0.35	n.a.	0.053	6.73/6.73	2/2	7.55/7.55	2/3
**MITOCHONDRIAL RESPIRATORY CHAIN**										
P40941	At5g13490	ADP,ATP carrier protein 2, mitochondrial	Unique in Col-0	Unique in Col-0	n.a.	n.a.	8.21/8.21	2/2	8.31/8.31	2/2
**METABOLISM**										
P55217	At3g01120	Cystathionine gamma-synthase 1, chloroplastic	mutant unique	mutant unique	n.a.	n.a.	6.36/7.77	2/2	7.82/6.93	2/2
Q9FIJ7	At5g47840	Adenylate kinase 2, chloroplastic	mutant unique	mutant unique	n.a.	n.a.	11.01/8.51	3/2	19.43/14.49	3/4
Q9LIR4	At3g23940	Dihydroxy-acid dehydratase, chloroplastic	Unique in Col-0	0.31	n.a.	0.04	7.31/7.59	2/2	6.09/6.09	3/5
Q8L940	At5g01410	Pyridoxal 5'-phosphate synthase subunit PDX1.3	1.49	1.85	0.053	0.018	23.65/23.94	6/6	29.77/29.77	28/28
Q9LEU8	At5g10920	Argininosuccinate lyase, chloroplastic	mutant unique	mutant unique	n.a.	n.a.	11.08/3.69	3/1	12.19/3.87	4/3
**UNKNOWN**										
Q8LDV3	At4g13200	Uncharacterized protein At4g13200, chloroplastic	Unique in Col-0	0.30	n.a.	0.021	3.43/9.91	1/3	7.57/22.70	3/5

*n.a, not applicable*.

1 *The sum of the ion scores of all peptides that were identified*.

2 *Total number of identified peptide sequences for the protein*.

3 *The percentage of the protein sequence covered by identified peptides*.

4 *Total number of identified peptide spectra matched for the protein*.

We therefore assign the differences in the proteome of the two presently studied mutants to their different molecular backgrounds. Indeed, the mutant *pld*α*1-1* has a tandem T-DNA insertion (our observation) in the 3rd exon close to the C-terminus while the mutant *pld*α*1-2* has one T-DNA insertion in the 2nd exon in the middle of the gene (Zhang et al., 2004; Bargmann et al., 2009). Consistent with this, very similar comparative proteomic study of proteomes of Arabidopsis mutants in *KATANIN1* gene showed differences between the mutants as well (Takáč et al., 2017).

Several bioinformatic tools were employed to functionally classify proteomes. First, we performed a GO annotation analysis, KEGG based classification and classification of protein domains in the whole proteomes of Col-0, *pld*α*1-1*, and *pld*α*1-2* mutants. These analyses did not reveal obvious differences between the analyzed lines (Figures S1–S4). Next, the DAPs commonly found in both mutants (as compared to the wild type) were subjected to similar bioinformatic analyses. GO annotation according to biological process showed that proteins involved in metabolic processes were quite abundant in the common differential proteomes of the mutants (Figure S5A). Further, proteins involved in the response to abiotic stimulus and developmental processes were also affected. Employing classification of DAPs by GO annotation according to cellular component, proteins localized to cytoplasm and plastids were the most abundant ones. In addition, vacuolar, mitochondrial and plasma membrane proteins were also affected (Figure S6A). Similar analysis according to molecular function showed overabundance of proteins binding to diverse molecules including organic compounds, ions, proteins as well as nucleotides (Figure S7A). In addition, we also performed a comparison of GO annotations of differential proteomes in both mutants separately. Consistently with the global proteome comparison, the differential proteomes showed also very similar patterns when subjected to bioinformatic analysis (Figures S5B, S6B, S7B).

**Figure 2:**
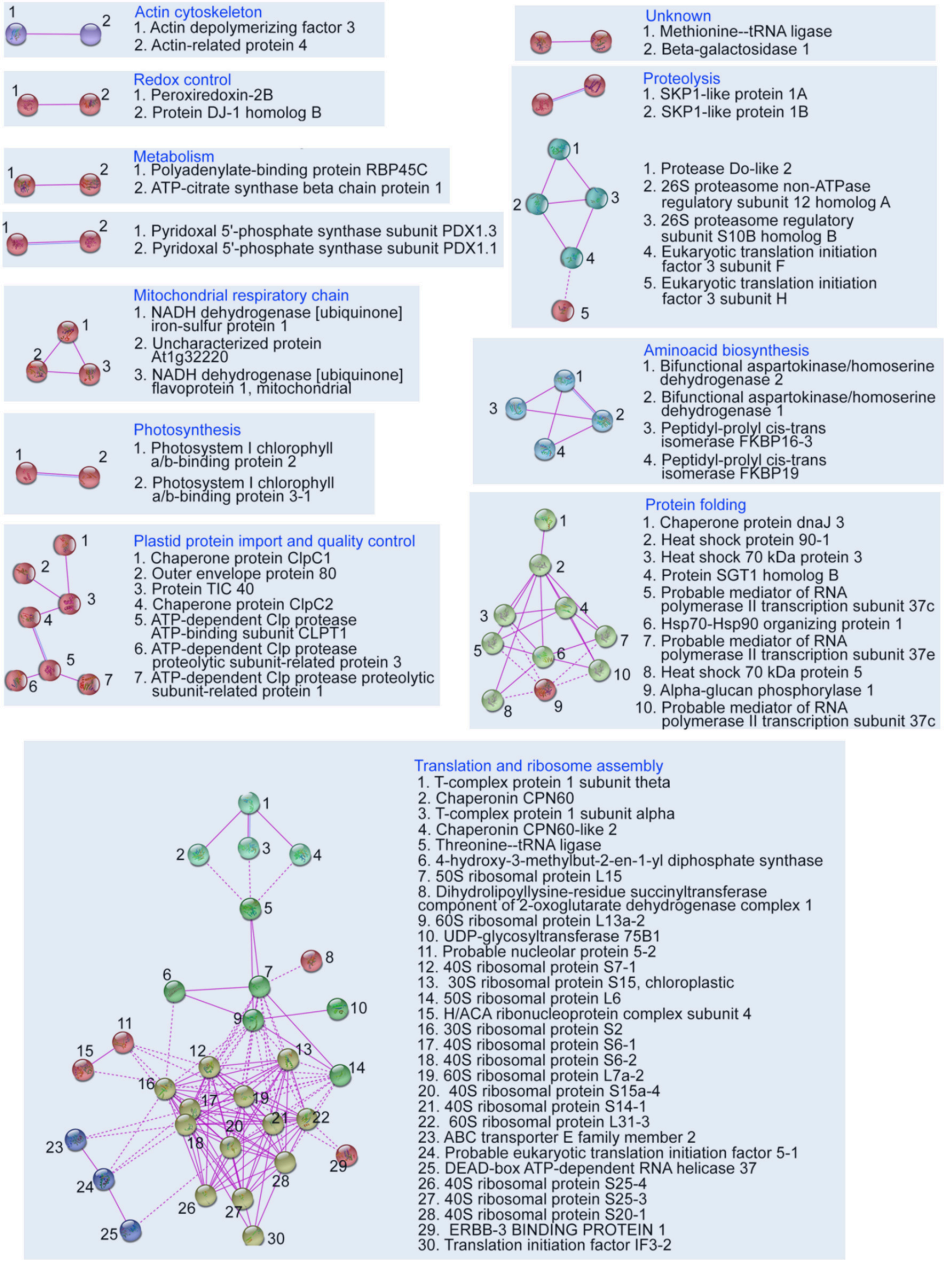
Depiction of protein interaction networks in combined differential proteome of both *pld*α*1* mutants as constructed using STRING web based application. Protein-protein interactions based on experimental evidence are considered. Note that interactions of homologous and heterologous proteins are also taken into account. The diagram was prepared using high confidence.

In the next step we attempted to reveal which protein complexes were affected in the *pld*α*1* mutants. Together, 12 protein clusters were found by STRING web-based application, employed to illustrate a protein network involving experimentally proved interactions among DAPs (Figure 2). Most abundant protein clusters were comprised of proteins involved in translation, cytoplasmic and plastidic ribosome biogenesis, heat shock proteins (HSPs), chloroplast protein import and quality control.

### Deregulation of Proteins Involved in Translation

**Figure 3:**
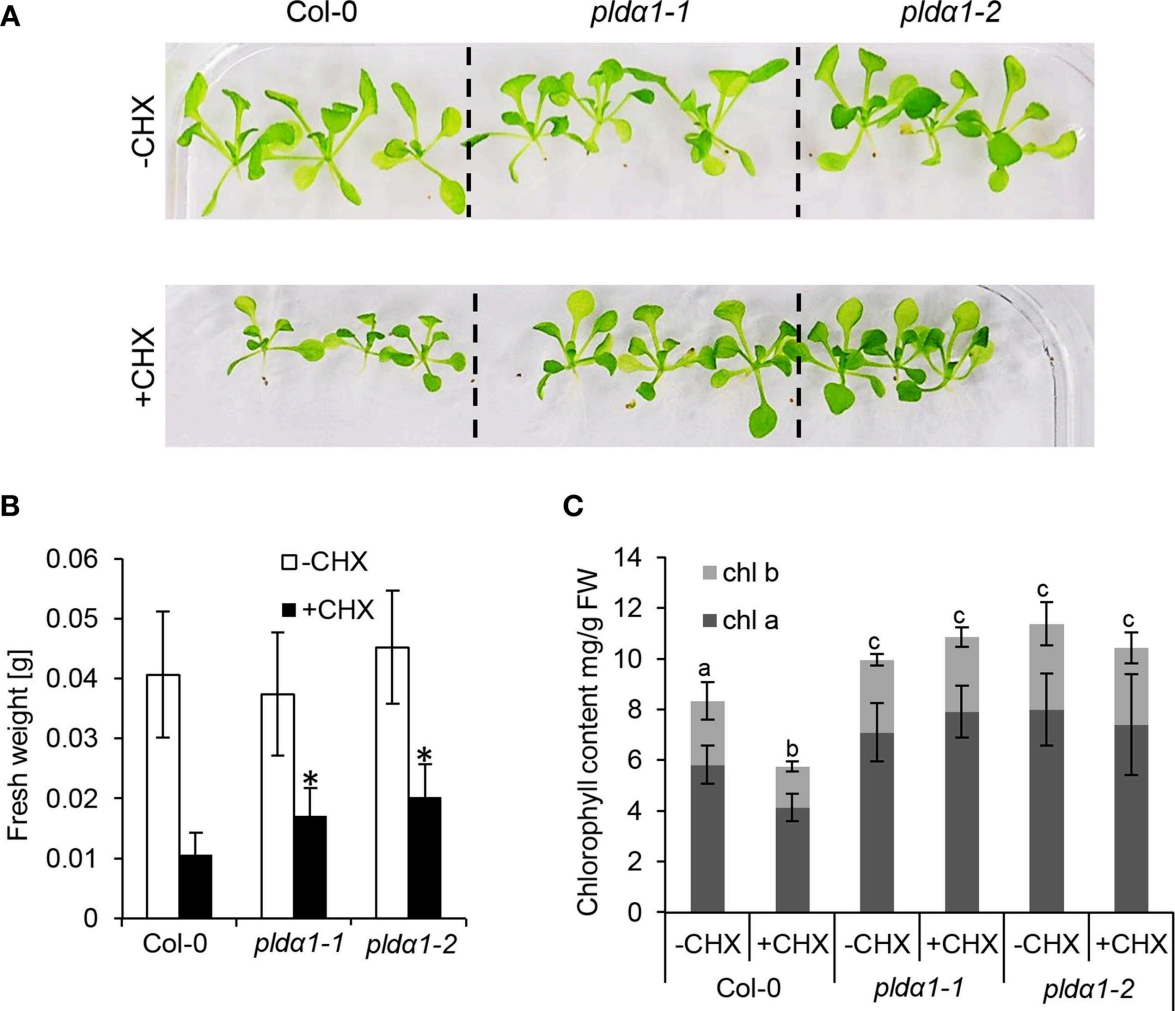
Examination of *pld*α*1* mutants sensitivities to the cycloheximide (CHX). **(A)** Pictures of representative seedlings of wild type Col-0 and *pld*α*1-1* and *pld*α*1-2* mutants grown on 1/2 MS medium (–CHX) or plants exposed to 0.4 μM CHX. **(B)** Graph showing average fresh weight of 16-days-old plants exposed (+CHX) or not exposed to 0.4 μM CHX (–CHX). ^*^ Indicate significant differences between mutants and wild type at *p* ≤ 0.05 according to one-way ANOVA test. Error bars represent standard deviations. **(C)** Graph showing chlorophyll *a* and *b* contents in *pld*α*1* mutants. Different letters above the bars indicate statistically significant differences at *p* ≤ 0.05 according to the one-way ANOVA test. Error bars represent standard deviations. FW = fresh weight.

Further analyses revealed robust alteration of proteins involved in translation in the mutants. Despite that only two such DAPs were found commonly in both mutants (Table 1), the differential proteomes of the individual mutants (Table S1) contained very similar set of proteins encompassing in numerous cases different isoforms of the same protein. These include translation initiation and elongation factors, tRNA ligases, and DEAD box RNA helicases (Table 1 and Table S1). Furthermore, altered abundance of both cytosolic and chloroplast ribosomal proteins were encountered. Among the chloroplast ones, three proteins (30S RPS15; 50S RPL15; 50S RPL6) were downregulated, while 30S RPS2 was upregulated in the mutants (Table S1). Altered translation is also suggested by changed abundances of proteins involved in mRNA processing (polyadenylate-binding protein RBP45C) and the processing of nascent proteins (nascent polypeptide-associated complex subunit alpha-like protein 2). Interestingly, both *pld*α*1* mutants show decreased levels of MORF2 (multiple site organellar RNA editing factor, designated also as RIP2), an important mRNA editing regulator. In this respect, abundances of another RNA editing regulatory proteins belonging to pentatricopeptide repeat (PPR) proteins were also significantly altered. Such results indicate that both cytosolic and plastidic translation machineries are altered in the *pld*α*1* mutants. In order to validate these data, the response of *pld*α*1* mutants and wild type plants to cycloheximide, an inhibitor of protein synthesis was evaluated. Both *pld*α*1* mutants exhibited increased tolerance to this inhibitor in terms of fresh weight (Figures 3A,B) as well as chlorophyll content (Figure 3C), thus confirming differences in protein translation between the wild type and *pld*α*1* mutants.

### Protein Folding

In addition to protein synthesis, we observed a robust alteration in protein folding. In general, decreased abundances of proteins involved in protein folding (Table 1 and Table S1) such as heat shock 70 kDa protein 5 (HSP70B), heat shock protein 90-1, and HSP70-HSP90 organizing protein 1 in *pld*α*1* mutants were observed. Similarly, chaperones of plastidic proteins (ClpC1, ClpC2, and ClpB3) were downregulated as well.

### Protein Degradation

Except for protein synthesis, disturbed levels of proteins involved in protein degradation by cytosolic proteasome complex, as well as plastidic proteases (Table 1 and Table S1) were found. Several components of the proteasome complex were deregulated, including proteasome subunit beta type-5-A, SCF family of E3 ubiquitin ligases SKP1A and SKP1B, protein SGT1 homolog B, and 26S proteasome non-ATPase regulatory subunit 12 homolog A. This suggests instability of proteasome complex in the *pld*α*1* mutants. Furthermore, members of Clp chloroplast protease system, including ClpR1, ClpR3, and ClpT1 proteases, were downregulated in the *pld*α*1* mutants. In addition, protease Do-like 2 and organellar oligopeptidase A, chloroplastic/mitochondrial were disturbed too. These data suggests that PLDα1 is possibly involved in the turnover of chloroplast and cytosolic proteins.

### Evaluation of Chloroplast Proteins and Proteins Carrying Chloroplast Targeting Signal Peptides

**Figure 4:**
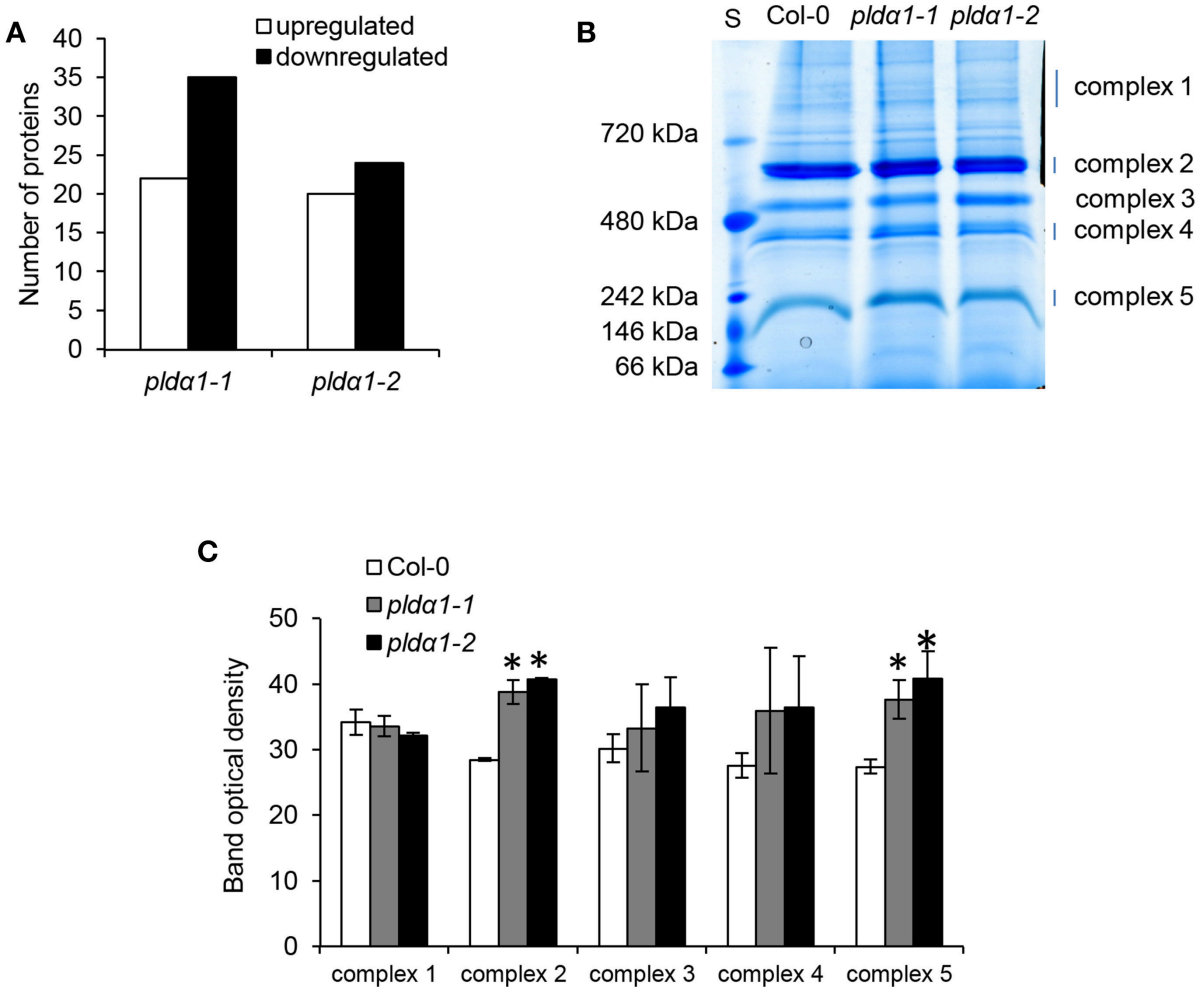
Abundances of chloroplastic proteins and complexes in the wild type Col-0 and in the *pld*α*1* mutants. **(A)** Graph showing numbers of proteins with chloroplast transit peptide (as predicted by ChloroP 1.1 server) found in differential proteomes of *pld*α*1* mutants. **(B)** Representative image of blue native electrophoretic gel prepared from membranes originating from isolated chloroplasts of wild type and *pld*α*1* mutants. S means molecular weight marker. **(C)** Quantification of band densities in **(B)**. ^*^ Indicate significant differences between mutants and wild type at *p* ≤ 0.05 according to the one-way ANOVA test. Error bars represent standard deviations.

In order to estimate abundances of plastidic proteins, differential proteomes of *pld*α*1* mutants were screened for proteins carrying chloroplast transit peptide. Together 57 and 44 such proteins were found in *pld*α*1-1* and *pld*α*1-2* mutants, respectively (Figure 4A, Table S2). The majority of these proteins showed decreased abundances in the mutants compared to wild type, indicating disturbed import of chloroplast proteins. Photosynthetic proteins are encoded by nuclear genome and following translation they are imported to the chloroplast for maturation and transported to their designated place. As revealed by our proteomic analysis, *pld*α*1* mutants exhibit significant changes in photosynthetic protein abundances (Table 1 and Table S1). While photosystem II 22 kDa protein was downregulated, photosystem I chlorophyll a/b-binding protein 2 was more than 6 times upregulated. Other photosynthetic proteins, such as light-harvesting complex-like protein 3 isotype 1, cytochrome b6-f complex iron-sulfur subunit, putative oxygen-evolving enhancer protein 2-2 and PR5-like protein 1A were also significantly upregulated in the mutants (Table 1 and Table S1). To validate altered abundances of photosynthetic proteins, we isolated chloroplasts from the above ground parts of wild type plants and *pld*α*1* mutants, and separated the digitonin-dissolved membranous chloroplast complexes using blue native electrophoresis (Figure 4B). In agreement with the above results, some complexes, particularly those with molecular weight close to 200 and 650 kDa, showed significantly higher abundances in the mutants when compared to the wild type (Figure 4C).

### Protein Import to Chloroplast

**Figure 5:**
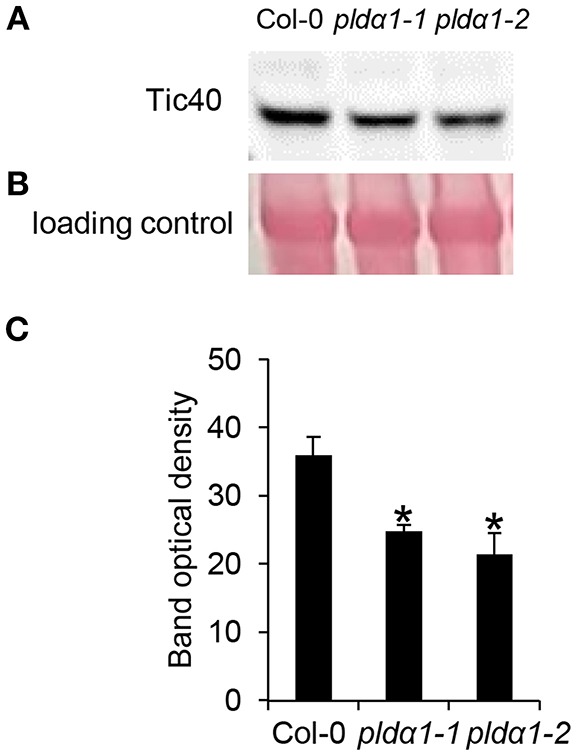
Immunoblotting analysis of TIC40 in above ground parts of wild type Col-0 and *pld*α*1* mutants. Images in **(A,B)** are sections from original membrane which is presented in Figure S8. **(A)** Immunoblot probed with anti-TIC40 antibody. **(B)** Visualization of proteins transferred on nitrocellulose membranes using Ponceau S. **(C)** Optical density quantification of respective band in **(A)**. ^*^ Indicate significant differences between mutants and wild type at *p* ≤ 0.05 according to the Student *t*-test. Error bars represent standard deviations.

Alteration of chloroplast protein import might be a consequence of decreased abundance of Tic40 (translocon at the inner) and HSP proteins (Table 1 and Table S1), both representing important components of the chloroplast import Tic complex (Kovacheva et al., 2005). Immunoblotting analysis using anti-Tic40 primary antibody validated the decreased abundance of this protein in both *pld*α*1* mutants (Figure 5). Image of the entire immunoblot is presented in Figure S8.

### Chloroplast Biogenesis

**Figure 6:**
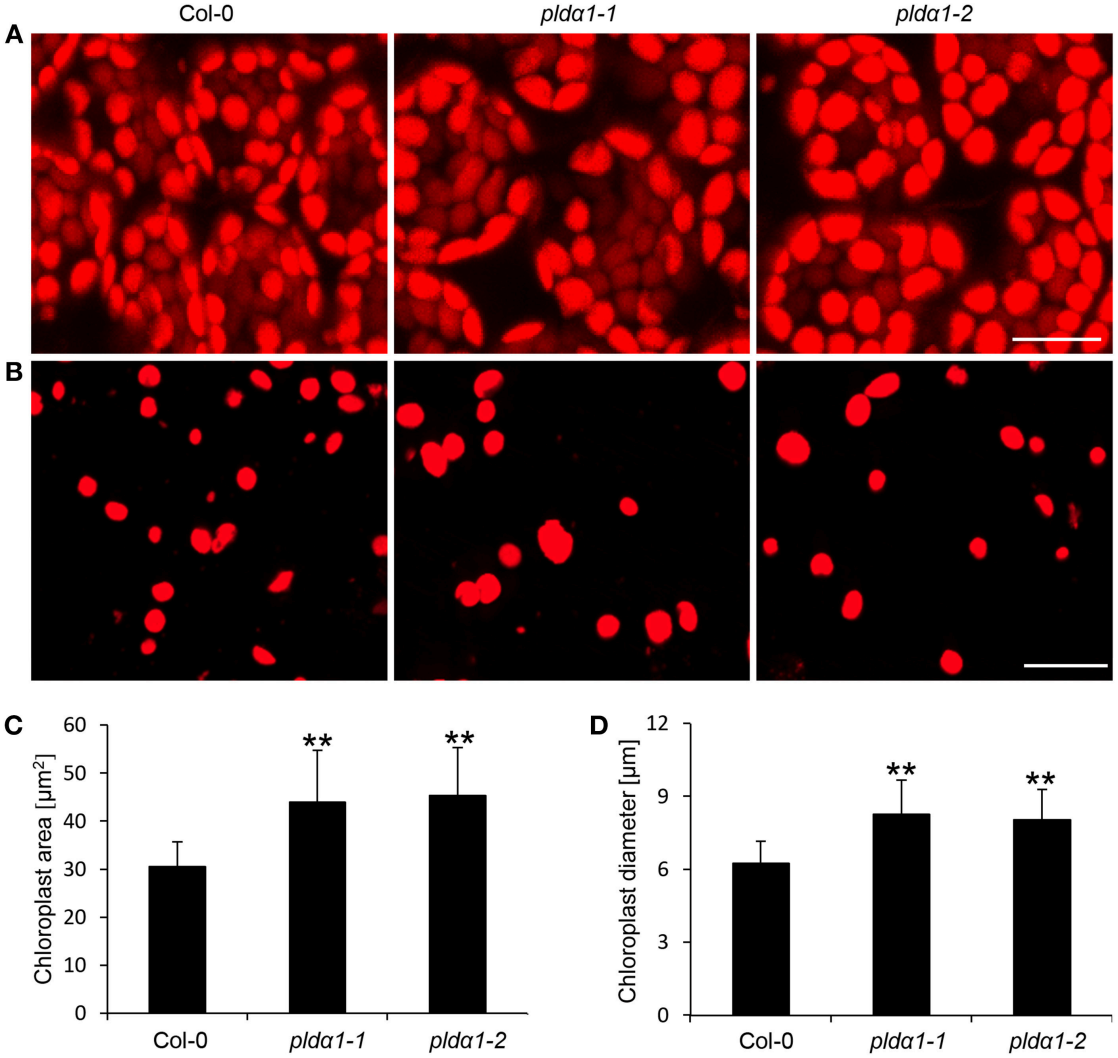
Chloroplast size in *pld*α*1* mutants and wild type Col-0. **(A)** Chloroplast size in leaf mesophyll cells of Col-0 and *pld*α*1* mutants. **(B)** Isolated chloroplasts from leaf cells of Col-0, *pld*α*1-1*, and *pld*α*1-2*. Note that chloroplasts in the *pld*α*1* mutants are bigger. Bars represent 20 μm. **(C,D)** Quantitative analysis of chloroplast area **(C)** and diameter **(D)** of chloroplasts in mesophyll cells of wild type Col-0 and both *pld*α*1* mutants. Final calculations were based on data collection from different leaf parts of 3 leaves (187–360 measured chloroplasts) obtained from 3 individual plants for each genotype. ^**^ Indicate significant differences between mutants and wild type at *p* ≤ 0.01 according to the Student *t*-test. Error bars represent standard deviations.

The disturbance in the import of chloroplast proteins may imply the altered chloroplast development and morphology in the *pld*α*1* mutants. To prove the disturbed morphology of chloroplasts, we quantitatively monitored the size of mesophyll chloroplasts, using chlorophyll autofluorescence observation *in vivo* by confocal microscopy (Figures 6A,B). The average areas and diameters of chloroplasts in both mutants were significantly bigger in comparison to the wild type (Figures 6C,D). Increased size of chloroplasts may also result in increased chlorophyll content in the mutants. In agreement, the chlorophyll content in the above ground parts was higher in both mutants, when compared to the wild type (Figure 3C). This is accompanied by deregulation of proteins important for chlorophyll biosynthesis, such as magnesium-chelatase subunit ChlI-1, magnesium-protoporphyrin IX monomethyl ester [oxidative] cyclase, and porphobilinogen deaminase in the *pld*α*1* mutants (Table 1 and Table S1).

## Discussion

PLDα1 exerts many essential functions in plants, depending also on its product PA, an important phospholipid signaling messenger. PLDα1 and PA are inevitable for abiotic stress responses, either through controlling ABA responses (Zhang et al., 2004, 2009b), MAPK signaling (Yu et al., 2010), or cytoskeleton organization (Zhang et al., 2012). PA produced by PLDα1 binds to NADPH oxidase and affects ROS production in response to ABA in guard cells (Zhang et al., 2009b) while it binds also to 3'-phosphoinositide-dependent kinase-1 (AtPDK1) and stimulates Arabidopsis AGC kinase AGC2-1 in order to control root hair development (Anthony et al., 2004). In addition, PA binds to 14-3-3 proteins and hampers their ability to activate plant plasma membrane H^+^-ATPase, a proton pump important for multiple physiological functions (Camoni et al., 2012). Of note, some PLDα1 functions are dependent on G-protein complex (Roy Choudhury and Pandey, 2017) and sphingosine kinase (Guo et al., 2012).

Genetically modified plants are often used for functional studies in plant research. Phenotypic, physiological, or biochemical features of mutants serve as indications of respective gene functions. These features are mirrored by various changes at the molecular level, including changed protein abundances which can be studied by quantitative differential proteomic analysis (Takáč and Šamaj, 2015; Takáč et al., 2017). In this study, we aimed to quantitatively compare proteomes of above ground parts of two Arabidopsis *pld*α*1* mutants with the wild type plant. The most intriguing finding is the massive change in abundances of proteins contributing to chloroplast biogenesis in the *pld*α*1* mutants.

### PLDα1 Is Involved in mRNA Editing and Translation

Arabidopsis *pld*α*1* mutants exert massive deregulation of both cytoplasmic and chloroplast translational machineries. Such regulatory functions related to translation were not assigned to PLDα1 so far. Translation initiation factor IF 3-2 (named also suppressor of leaf variegation 9), which specifically controls the translation of chloroplast proteins (Zheng et al., 2016), shows increased abundance in the *pld*α*1-1* mutant. The impaired translation of chloroplast proteins is also indicated by changed abundances of chloroplast ribosomal proteins and proteins implicated in chloroplast ribosome biogenesis, such as chloroplast RH3 DEAD box RNA helicase (Asakura et al., 2012), or 4-hydroxy-3-methylbut-2-en-1-yl diphosphate synthase (AtcpRRF), a chloroplast ribosome recycling factor. AtcpRRF is responsible for the release of ribosomes from messenger RNA at the termination of chloroplast protein biosynthesis (Wang et al., 2010).

In flowering plants, mRNA editing occurs in mitochondria and chloroplasts, and might lead to changes in protein abundances. It can correct deleterious mutations, and in some cases it is required for plant viability (Shikanai, 2015). Most crucial molecular players responsible for mRNA editing are the MORF and PPR-containing proteins (Takenaka et al., 2012). Silencing of MORF2 in Arabidopsis leads to the reduction in editing of 22 plastid sites and 21 mitochondrial editing sites (Bentolila et al., 2013). One of these genes encodes chloroplastic ATP synthase subunit b (Bentolila et al., 2013), which is more than 3-fold upregulated in the *pld*α*1-2* and is specifically present in the *pld*α*1-1* mutant. The RNA editing function of MORF2 is modulated by interaction with porphobilinogen deaminase HEMC (Huang et al., 2017), which is also downregulated in *pld*α*1-1* mutant. It catalyzes the biosynthesis of uroporphyrinogen III, the central precursor of the chlorophyll biosynthesis (Shoolingin-Jordan, 1995), which is enhanced in the mutants as it was shown in our results. This points to the possible link between chlorophyll biosynthesis, RNA editing, and PLDα1. PPR-containing protein At1g02150 which accumulated in the *pld*α*1-2* mutant, belongs to the P subfamily of PPRs, which have RNA editing functions (Doniwa et al., 2010). In addition, RNA-binding protein CP31B (downregulated in *pld*α*1-1* mutant) is known to control multiple editing sites in plastid mRNA (Tillich et al., 2009). In summary, these data suggest an important new role of PLDα1 in mRNA editing.

### Chloroplast Protein Import Machinery and Quality Control Require PLDα1

The general import of plastidic proteins is mediated through distinct outer and inner envelope membrane protein complexes, called translocons at the outer and the inner envelope membrane of chloroplasts (TOC and TIC), respectively (Kovács-Bogdán et al., 2010). Nucleus encoded chloroplast proteins are translated on cytosolic ribosomes, and the nascent proteins are translocated into chloroplasts with the aid of HSP70 and HSP90 chaperones and HSP70-HSP90 organizing protein 1 (HOP1) co-chaperone (Clément et al., 2011; Fellerer et al., 2011) to the TOC and TIC translocons. Above we mentioned that *pld*α*1* mutants are compromised in cytosolic ribosome biogenesis and translation, indicating that the chloroplast protein translation might be impaired as well. Since HSP90 and HSP70-5 are strongly downregulated while HOP1 is upregulated, it is likely that the mutants are impaired in their transport toward TIC and TOC complexes. TIC complex, comprised of eight components including TIC40 (downregulated in *pld*α*1* mutants) is responsible for preprotein translocation across the inner envelope (Nakai, 2015). ClpC chaperones (ClpC1 and 2, both downregulated in the *pld*α*1-1* mutant) interact with TIC, and they affect the protein import to chloroplasts. Loss of ClpC1 or TIC40, results into lower protein import rates into isolated chloroplasts (Kovacheva et al., 2005). Recent study highlighted the role of ClpC chaperones and Clp proteolytic core (decreased abundances of CLPR1 and 3) in quality control during chloroplast import (Flores-Pérez et al., 2016). Next proteomic evidence about alteration of chloroplast protein import in the mutants is provided by the downregulation of outer envelope protein 80 (OEP 80). OEP 80 may be required for insertion of beta-barrel proteins in the outer membrane (Patel et al., 2008).

### PLDα1 Contributes to G-Protein Mediated Control of Leaf Variegation

Leaf variegation, an appearance of white leaf sectors, is caused by defective AtFtsH2/VAR2 plastid metalloprotease (Kato et al., 2007). Such albinotic leaf parts are impaired in chloroplast biogenesis (Kato et al., 2007). We have found several indications of possible involvement of PLDα1 in leaf variegation, as supported by increased abundance of translation initiation factor IF3-2 in the *pld*α*1-1* mutant. This protein acts as a suppressor of variegation (Zheng et al., 2016). Splice site mutation in ClpC2 and consequent reduced ClpC2 protein accumulation has been shown to suppress *var2* variegation (Park and Rodermel, 2004). This protein has lower abundance in the *pld*α*1-1* mutant. Leaf variegation was also connected to misbalance in plastid and cytosolic translation (Wang et al., 2018). Inhibition of chloroplast development by ATP-dependent zinc metalloprotease Ftsh in Arabidopsis is suppressed by activation of the heterotrimeric GPA1 (Zhang et al., 2009a), which is an interactor of PLDα1 (Zhao and Wang, 2004). It is known that GPA1 alleviates PLDα1 activity (Zhao and Wang, 2004). Recently, it was reported that PLDα1 protein is a key component and modulator of the G-protein complex (Roy Choudhury and Pandey, 2016). Based on these results, PLDα1 may play a role in G protein complex—mediated control of leaf variegation.

### PLDα1 Integrates Translation, RNA Editing, Chloroplast Protein Import, and Proteolysis in Order to Keep Homeostasis During Chloroplast Biogenesis

Intriguingly, the above mentioned defects found in *pld*α*1* mutants are important determinants of chloroplast biogenesis. Suppression of translation initiation factor IF 3-2 (downregulated in the mutants) has a negative effect on chloroplast biogenesis in Arabidopsis (Zheng et al., 2016). Homeostasis in proteins controlling plastid protein translation (Pogson and Albrecht, 2011) and mRNA editing in chloroplast are also important for chloroplast biogenesis (Ramos-Vega et al., 2015). Deficiency in the ATP-dependent Clp protease proteolytic subunit-related protein 1 (ClpR1, downregulated in the mutants) leads to the lower accumulation of plastome-encoded proteins, delayed plastid ribosome assembly and defects in chloroplast biogenesis (Koussevitzky et al., 2007; Olinares et al., 2011). CLPB3 is also a molecular chaperone involved in plastid differentiation and in mediating internal thylakoid membrane formation (Myouga et al., 2006). Proper chloroplast biogenesis depends on balanced chloroplast protein import, translation, degradation, and quality control (Jarvis and López-Juez, 2013). In concert with these data, the abundance of chloroplast protein complexes in the mutants was also changed. Therefore, PLDα1 seems to integrate regulation of these processes in order to control chloroplast biogenesis. Significant part of the DAPs involved in chloroplast biogenesis, including HSPs and TIC40 are also involved in stress response. Accordingly, the defects in PA production in the *pld*α*1* mutants likely mimic the abiotic stress conditions.

A direct mechanism of translational regulation by PLDα1 is unknown. In a recent proteomic study, several cytosolic ribosomal proteins were identified as PA targets (McLoughlin et al., 2013). It suggests that PLDα1 may affect the translation via its PA producing capability. Another possible mechanism might require PLDα1 interaction with and modulation of G protein complex (Gookin and Assmann, 2014; Pandey, 2016; Roy Choudhury and Pandey, 2016). In order to evaluate this possibility, we prepared an intersection between the current differential proteome of *pld*α*1* mutants and interaction partners of G-protein complex found by high throughput yeast two hybrid assay (Klopffleisch et al., 2011) (Table 2). Two proteins directly interacting with G-complex subunits and other 8 interacting with primary binding partners of the subunits were found. Proteins involved in translation have been found to bind primary interacting partners of G-protein complex subunit. This shows that PLDα1 may affect the translational machinery through PA or by crosstalk with G-protein complex signaling.

**Table 2:** Proteins known as primary or secondary interaction partners of G-protein complex.

**Sequence ID (TAIR)**	**Sequence ID (UNIPROT)**	**Sequence name**	***pldα1-1*/Col-0 ratio**	***pldα1-2*/Col-0 ratio**	**Bait locus (Klopffleisch et al., 2011)**	**Bait name (Klopffleisch et al., 2011)**
**At2g44350**	**P20115**	**Citrate synthase 4, mitochondrial**	**3.53**		**At3g26090**	**RGS1**
**At5g64120**	**Q43387**	**Peroxidase 71**		**0.50**	**At3g26090**	**RGS1**
At5g14320	P42732	30S ribosomal protein S13		0.23	At2g03670	CDC48B
At1g30230	P48006	Elongation factor 1-delta 1		0.55	At4g17730	SYP23
					At4g14716	ATARD1
					At5g65210	TGA1
At5g10360	P51430	40S ribosomal protein S6-2	0.56		At5g65210	TGA1
At5g59880	Q9ZSK4	Actin-depolymerizing factor 3		0.62	At5g65210	TGA1
At3g59760	Q43725	Cysteine synthase, mitochondrial	0.26		At4g14716	ATARD1
At3g15356	Q9LJR2	Lectin-like protein LEC	4.23		At4g14716	ATARD1
At4g18480	P16127	Magnesium-chelatase subunit ChlI-1, chloroplastic	0.53	0.52	At5g56750	NDL1
At1g78380	Q9ZRW8	Glutathione S-transferase U19		0.51	At3g18130	RACK1C

*Primary interaction partners of G-protein complex subunits are indicated in bold text (Klopffleisch et al., 2011)*.

Despite our findings on proteome and microscopic levels, *pld*α*1* mutants did not exhibit obvious differences in leaf color if compared to the wild type. It is likely caused by the relatively small extent of these differences. We assume that the PLDα1 deficiency causes an accumulation of proteins stabilizing the chloroplasts under such conditions. Such hypothesis is supported by the ability of the mutants to keep elevated chlorophyll contents during their exposure to cycloheximide.

This combined genetic, proteomic and biochemical study reports about new functions of PLDα1 which integrate cytosolic and plastidic translations, plastid protein degradation, and protein import into chloroplast in order to regulate chloroplast biogenesis in Arabidopsis.

## Availability of Data and Materials

The mass spectrometry proteomics data are available as Supplementary Material and have been deposited to the PRIDE (Vizcaíno et al., 2016) proteomic repository with the dataset identifier PXD009725 (http://www.ebi.ac.uk/pride/archive/). All pertinent “raw” data files and the “msf” results files are available to download. They can be viewed free of charge using Proteome Discoverer demo/viewer (http://planetorbitrap.com/demo-download). The datasets generated and analyzed within the remaining experiments are available from the corresponding author on reasonable request.

## Author Contributions

JŠ conceived and coordinated the experiments and helped to evaluate data. TT, TP, and OŠ made analyses and experiments. TT, JŠ, and OŠ wrote the manuscript. All authors reviewed the manuscript.

### Conflict of Interest Statement

The authors declare that the research was conducted in the absence of any commercial or financial relationships that could be construed as a potential conflict of interest.

## Supplementary Material

Data Sheet 1 Supplementary figures and tables.

Data Sheet 2 Identification and quantification-related details of differentially abundant proteins identified in *pld*α*1-1* mutant as compared to WT.

Data Sheet 3 Summary of protein characteristics and their relative abundances pertinent to differentially abundant proteins identified in *pld*α*1-2* mutant as compared to WT.

Data Sheet 4 Identification and quantification-related details of differentially abundant proteins identified in *pld*α*1-2* mutant as compared to WT.

Data Sheet 5 Summary of protein characteristics and their relative abundances pertinent to differentially abundant proteins identified in *pld*α*1-1* mutant as compared to WT.

## Abbreviations

ABA: abscisic acid
DAP: differentially abundant proteins
FDR: false discovery rate
GO: gene ontology
KEGG: Kyoto Encyclopedia of Genes and Genomes
MAP65-1: microtubule associated protein 65-1
MORF2: multiple site organellar RNA editing factor
TIC: translocon at the inner envelope membrane of chloroplasts
TOC: translocon at the outer envelope membrane of chloroplasts
PPR: pentatricopeptide repeat
PA: phosphatidic acid
PLDα1: phospholipase D alpha 1.
