# Supplementary material for: Proteomic Analysis of Arabidopsis pldα1 Mutants Revealed an Important Role of Phospholipase D Alpha 1 in Chloroplast Biogenesis
Source: Front Plant Sci. 2019 Feb 18;10:89. doi: 10.3389/fpls.2019.00089 (PMC6388422; doi:10.3389/fpls.2019.00089)
Supplement: Data Sheet 1 — Supplementary figures and tables. [file Data_Sheet_1.PDF]

frontiers

Supplementary Material

Proteomic analysis of Arabidopsis *pldα1* mutants revealed an important role of phospholipase D alpha 1 in chloroplast biogenesis

Tomáš Takáč, Tibor Pechan, Olga Šamajová, Jozef Šamaj*

*** Correspondence:** Jozef Šamaj: jozef.samaj@upol.cz

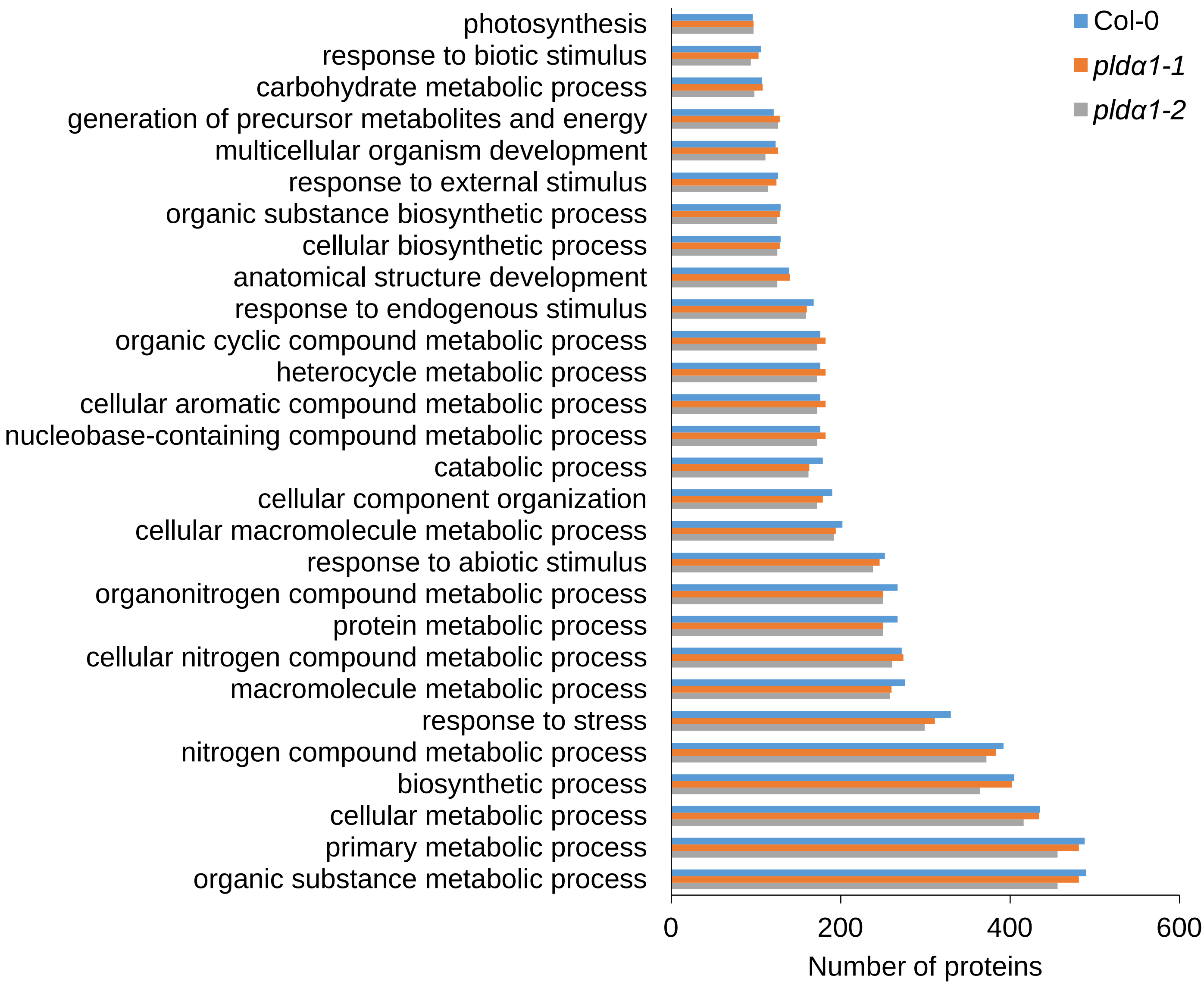

Figure S1. Classification of whole proteomes of Col-0, *pldα1-1* and *pldα1-2* mutant above ground parts using gene ontology annotation analysis (at 3rd level of ontology) in terms of biological process.

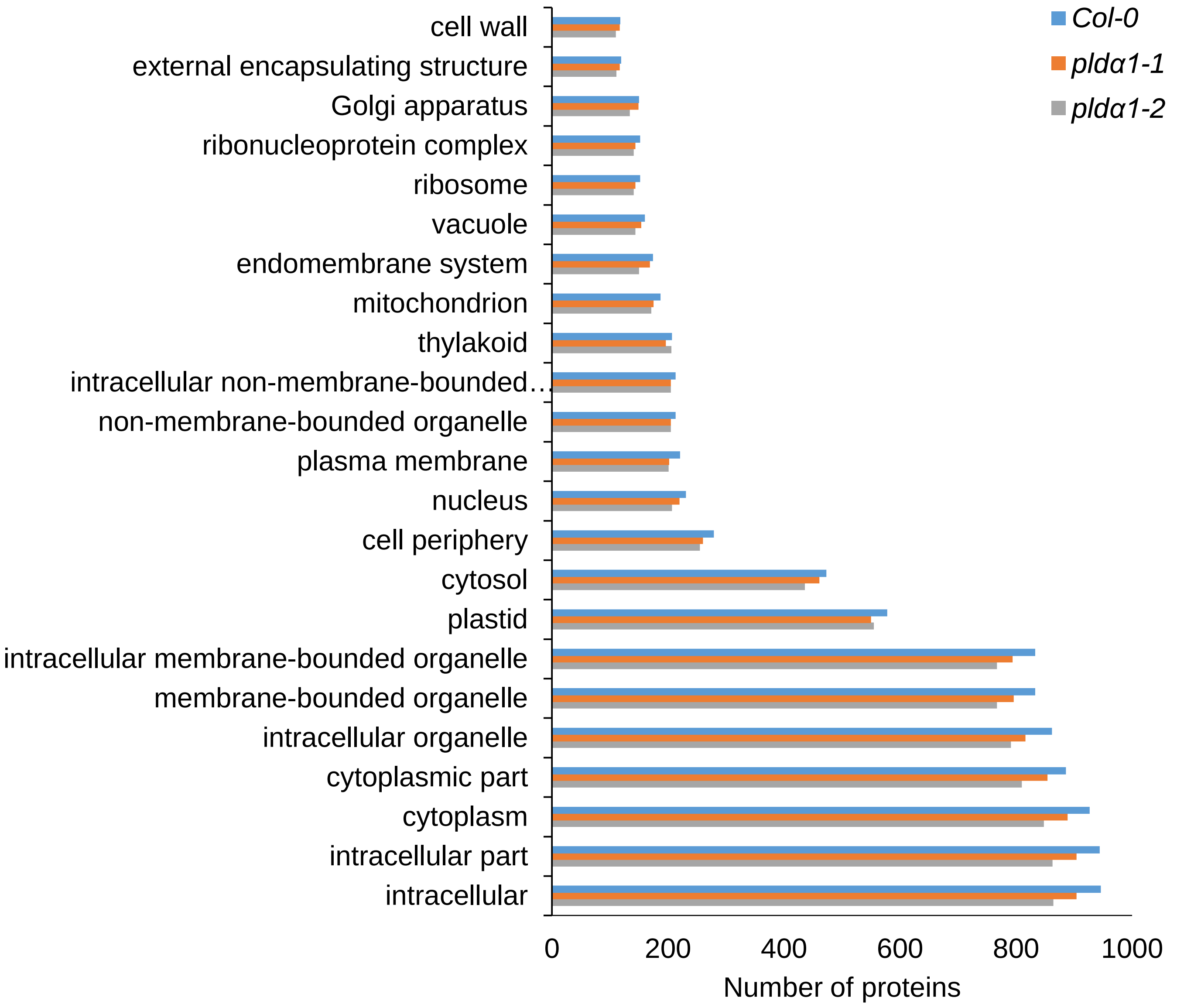

Figure S2. Classification of whole proteomes of Col-0, *pldα1-1* and *pldα1-2* mutant above ground parts using gene ontology annotation analysis (at 3rd, 4th and 5th levels of ontology) in terms of cellular compartment.

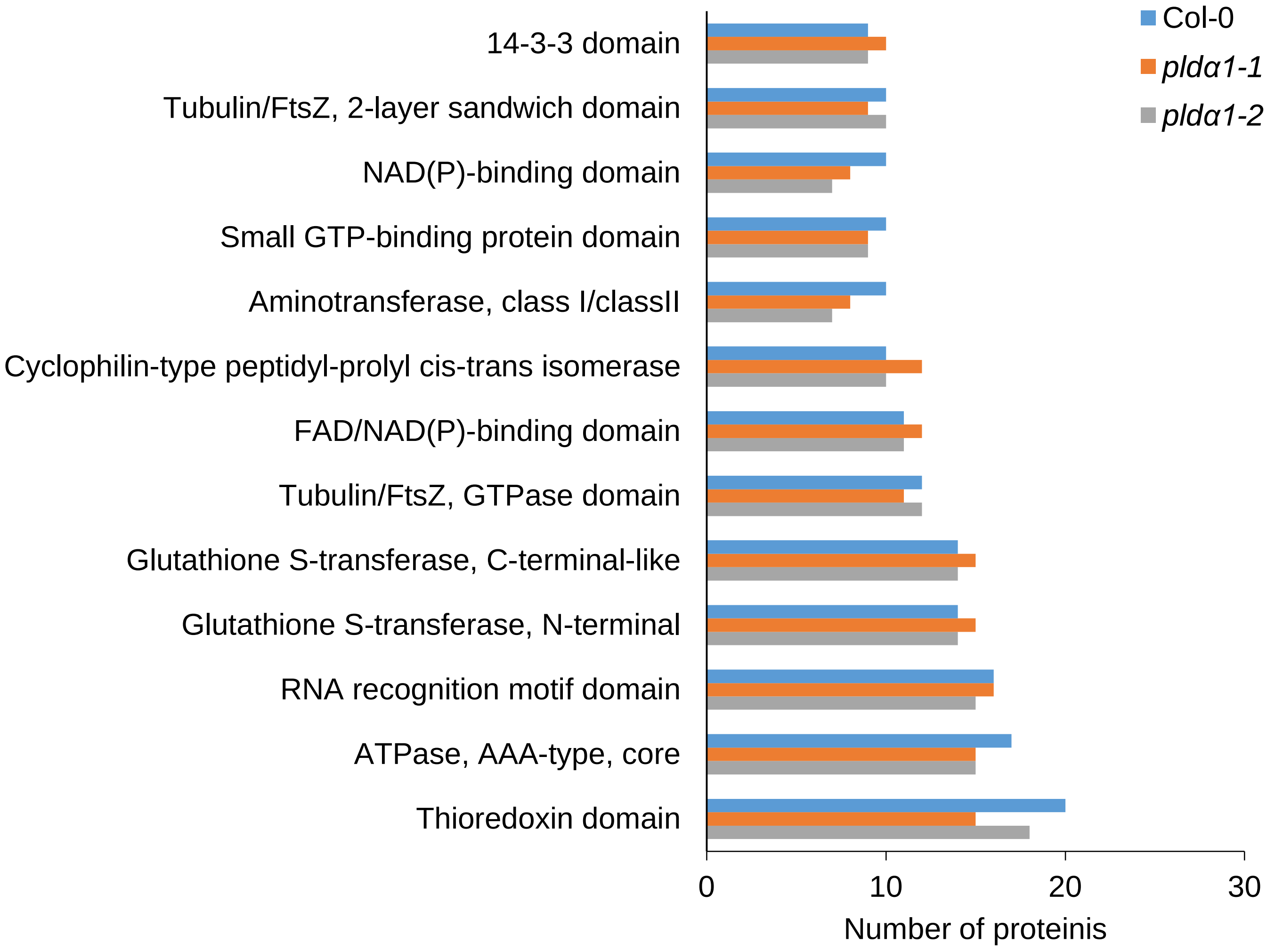

Figure S3. Distribution of protein domains in the whole proteomes of Col-0, *pldα1-1* and *pldα1-2* mutants as evaluated by Blast2Go software by using InterPro database.

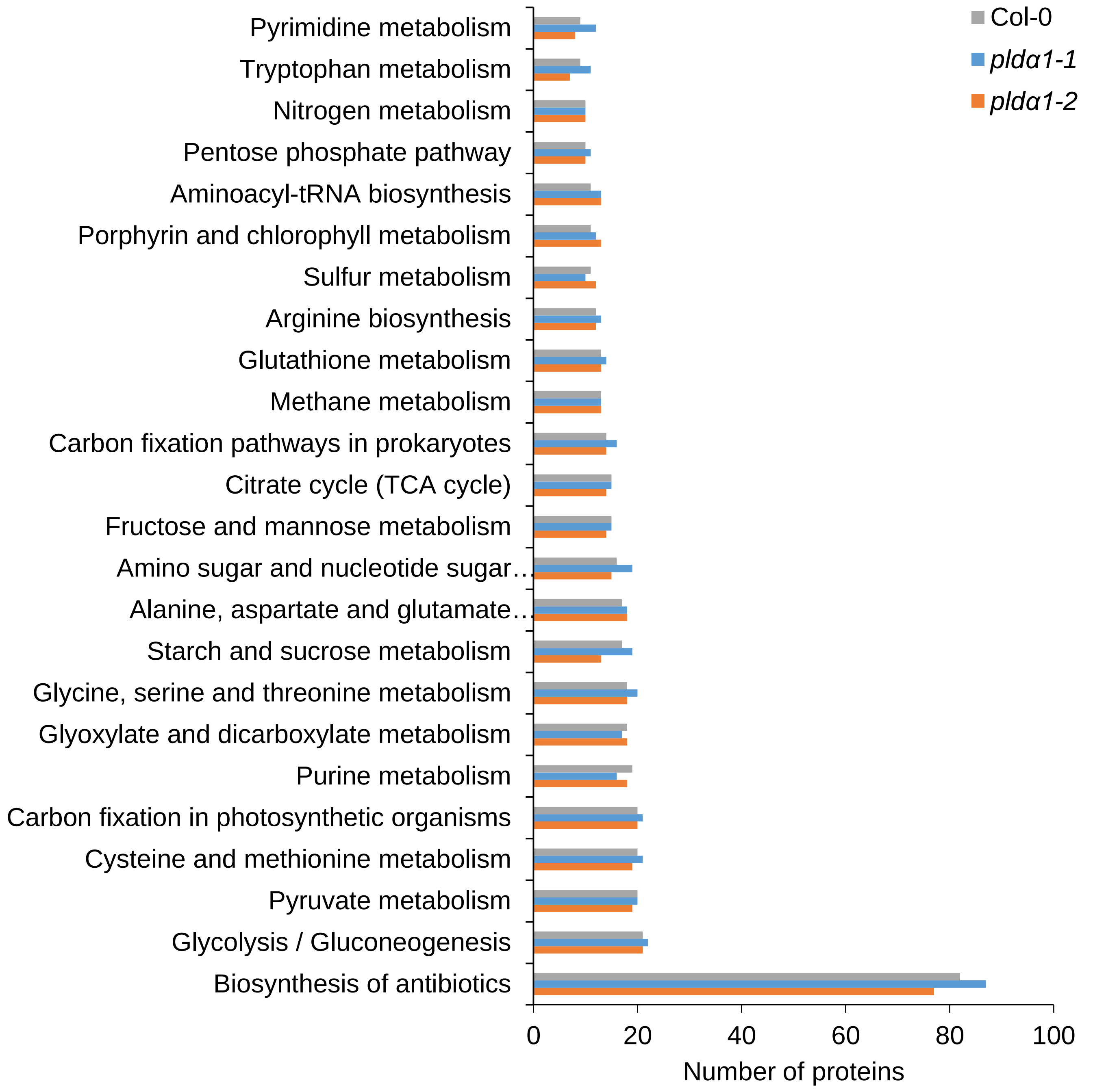

Figure S4. Classification of whole proteomes of Col-0, *pldα1-1* and *pldα1-2* mutant above ground parts using KEGG pathway analysis as evaluated by Blast2Go software.

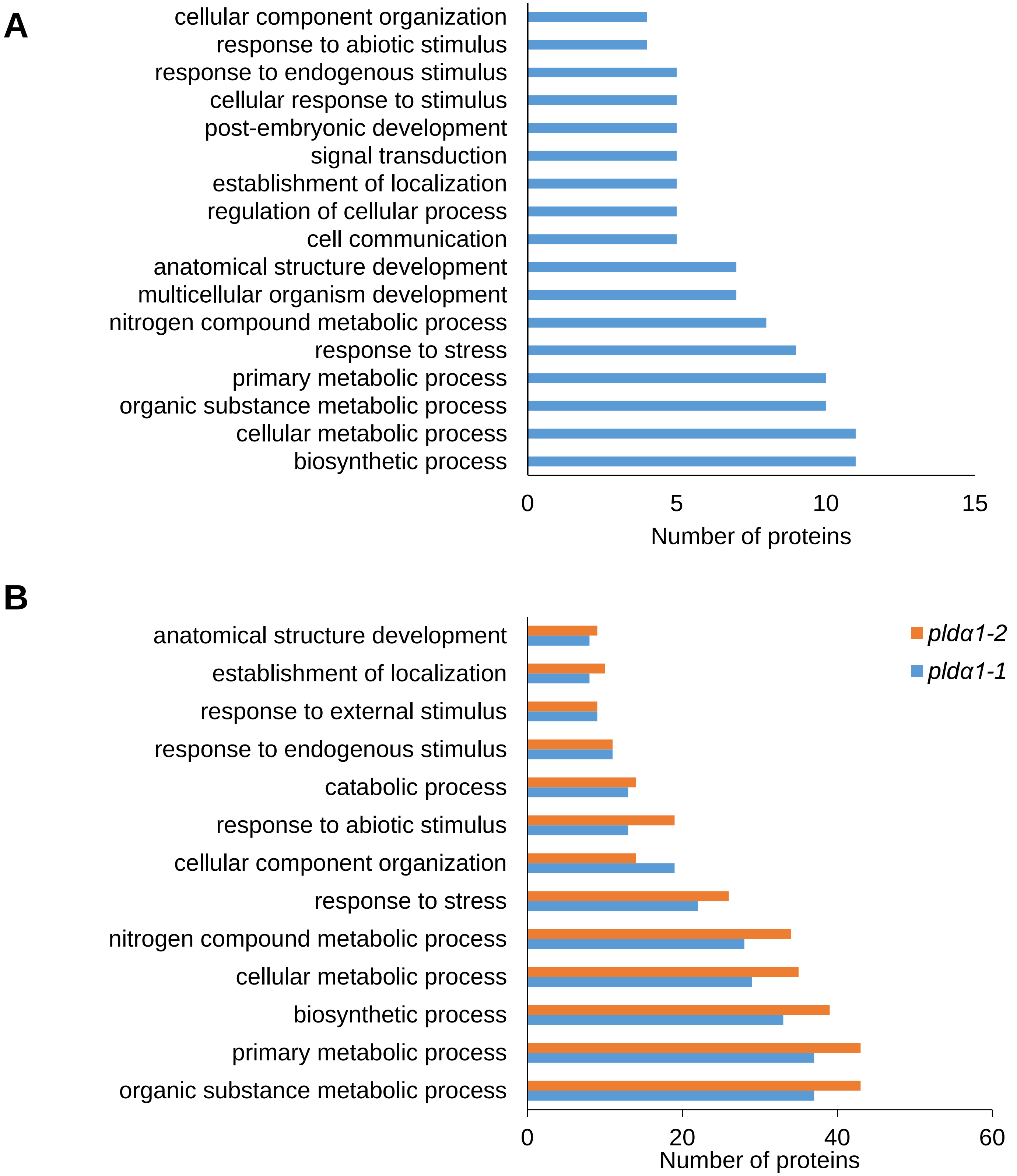

Figure S5 Classification of differential proteomes found in above ground parts of *pldα1-1* and *pldα1-2* mutants (as compared to wild type) using GO annotation analysis according to biological process (third level of ontology). (A) Graph showing the GO annotation of differentially regulated proteins found commonly in both mutants, (B) Graph showing the GO annotation of differentially regulated proteins found in both mutants separately.

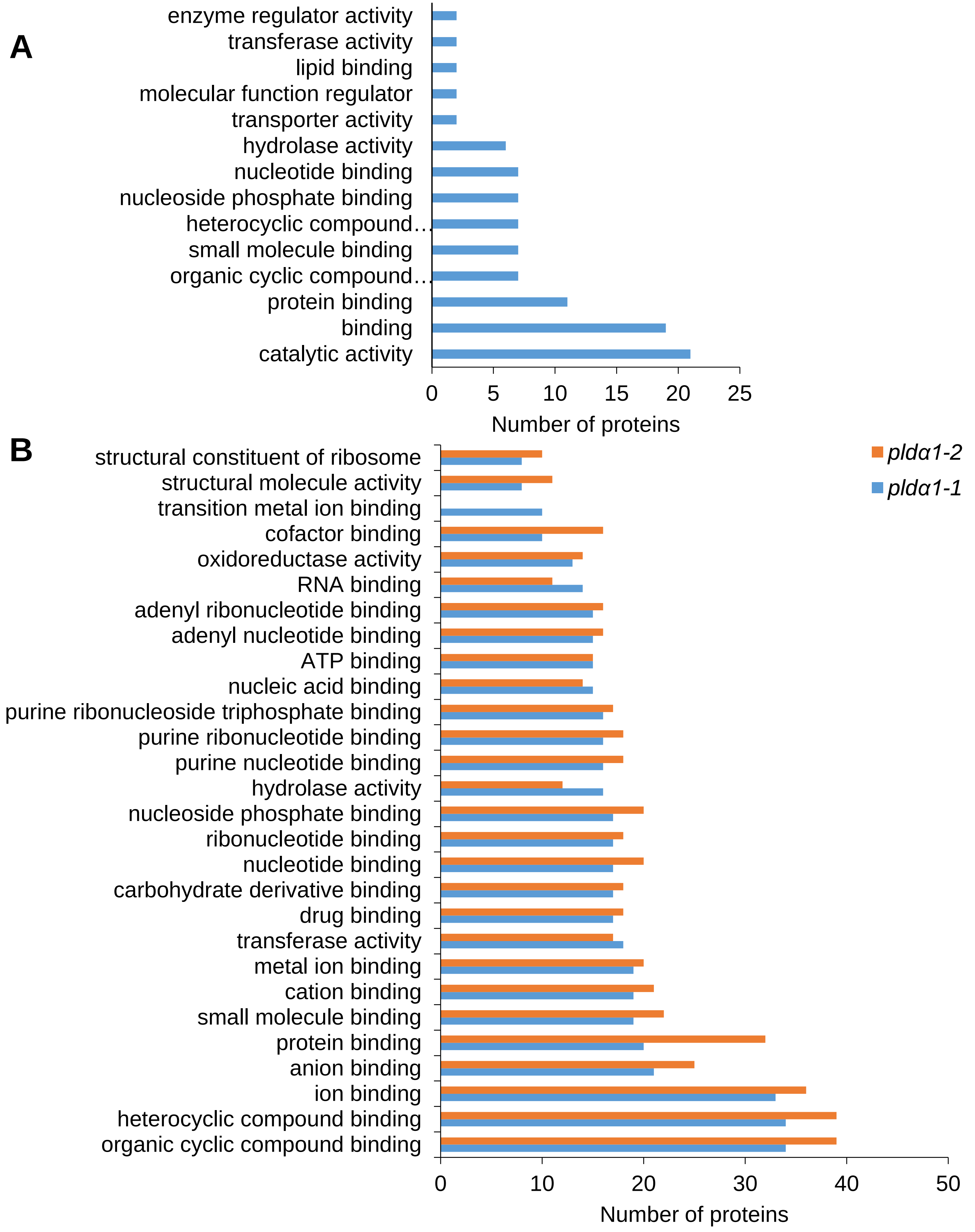

Figure S6 Classification of differential proteomes found in above ground parts of *pldα1-1* and *pldα1-2* mutants (as compared to wild type) using GO annotation analysis according to molecular function (third level of ontology). (A) Graph showing the GO annotation of differentially regulated proteins found commonly in both mutants, (B) Graph showing the GO annotation of differentially regulated proteins found in both mutants separately.

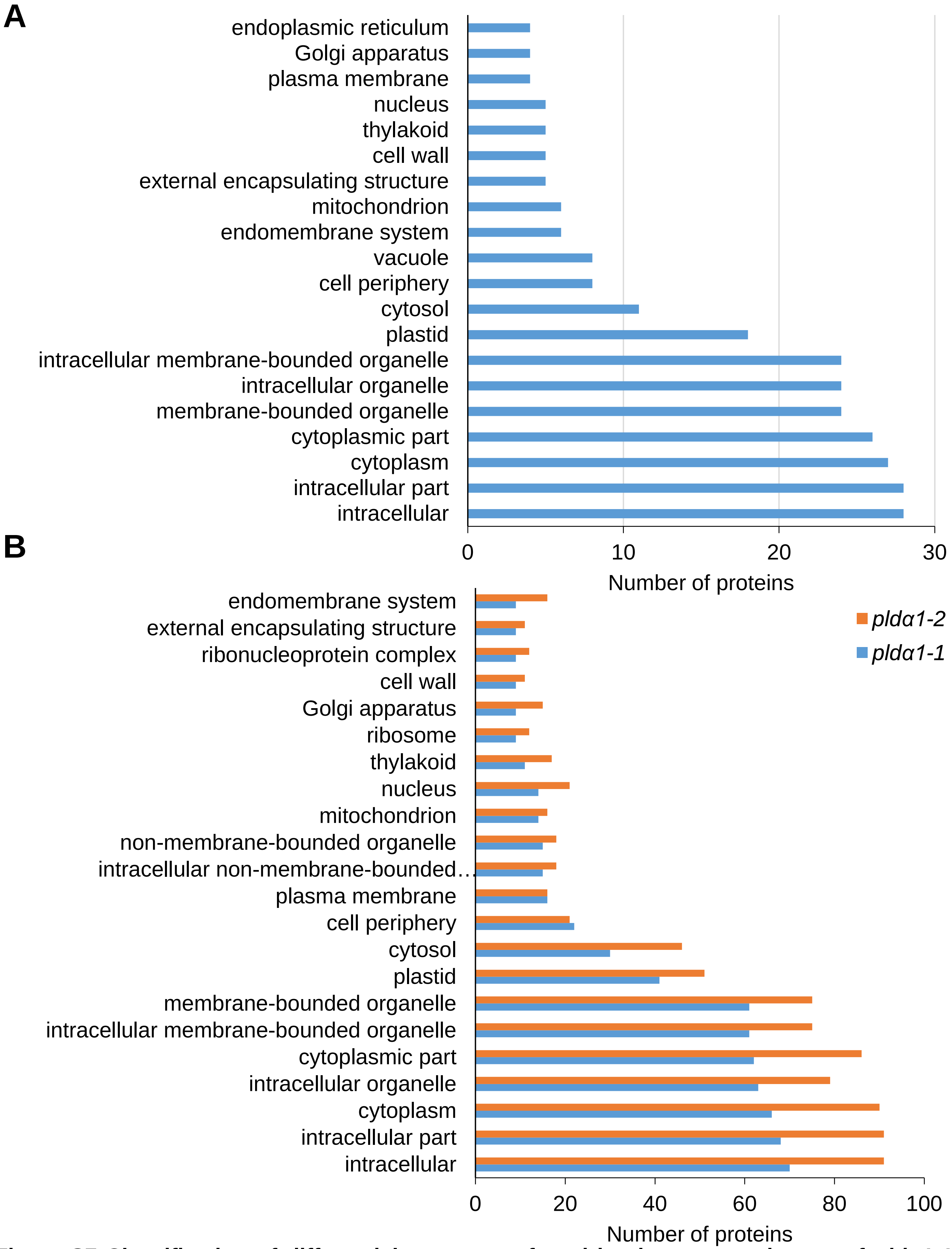

Figure S7 Classification of differential proteomes found in above ground parts of *pldα1-1* and *pldα1-2* mutants (as compared to wild type) using GO annotation analysis according to cellular compartment (third, fourth and fifth levels of ontology). (A) Graph showing the GO annotation of differentially regulated proteins found commonly in both mutants, (B) Graph showing the GO annotation of differentially regulated proteins found in both mutants separately.

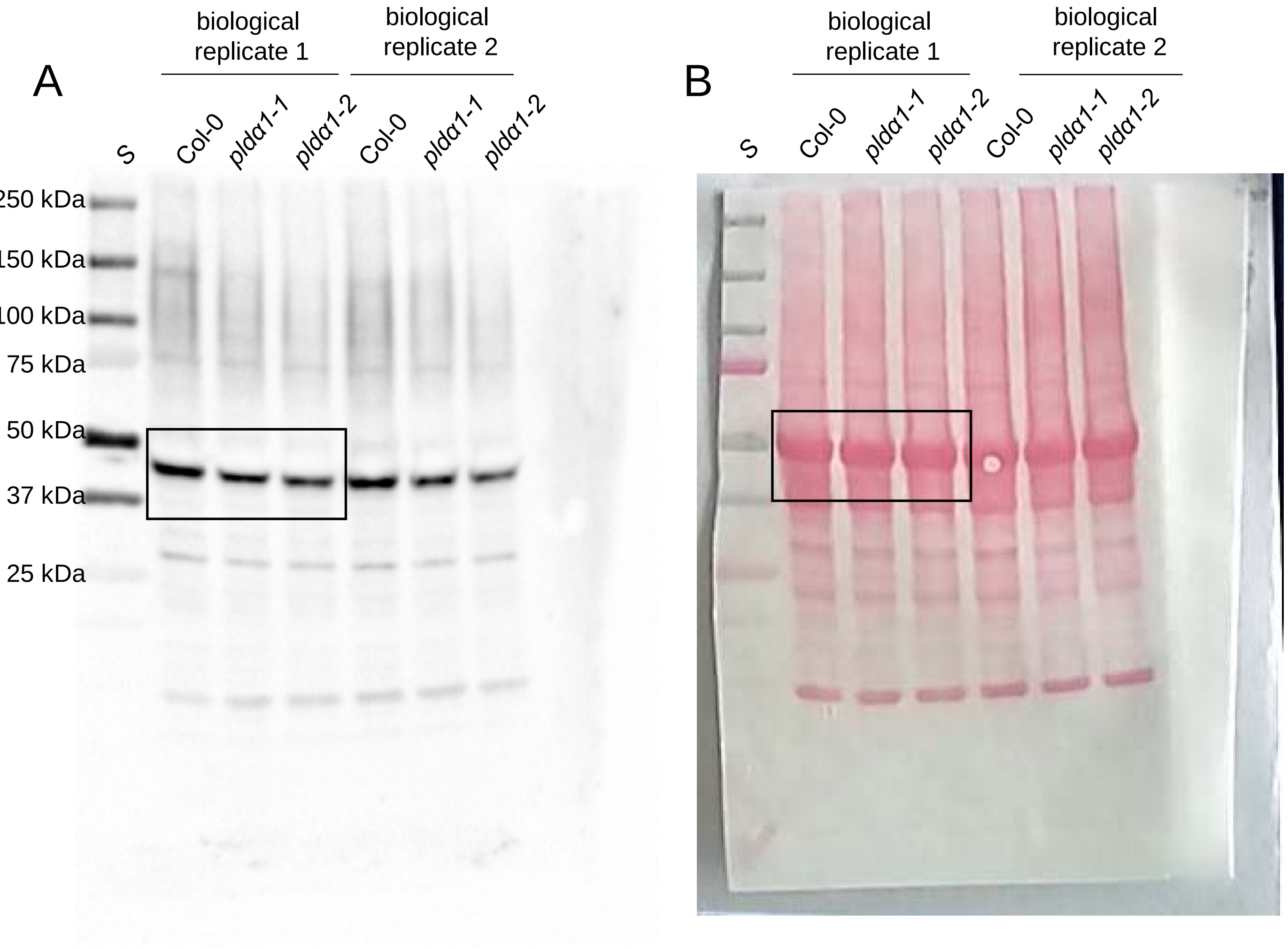

Figure S8 Immunoblotting analysis of TIC40 in *pldα1-1* and *plda1-2* mutants. Full scan of the entire original immunoblot presented in Figure 5A. (A) Entire membrane with chemiluminiscent signal observed after probing with anti-TIC40 antibody. The highlighted region shows the section presented in Figure 5A. (B) Full image of the respective membrane after staining with Ponceau S. The highlighted region shows the section presented in Figure 5B.

Table S1. Quantification details of proteins identified in above ground parts of Arabidopsis *pldα1-1* and *pldα1-2* mutant seedlings. n.e.v. = not enough values; n.d. = not detected; n.a. = not applicable;

Accession			fold change		*p* value					
TAIR	UNIPROT	Sequence Name	*pldα1-1* vs Col-0	*pldα1-2* vs Col-0	*pldα1-1* vs Col-0	*pldα1-2* vs Col-0	Total Score* *pldα1-1/ pldα1-2*	Total Peptides* *pldα1-1/ pldα1-2*	Total % Seq Coverage* *pldα1-1/ pldα1-2*	Total Spectra* *pldα1-1/ pldα1-2*
		**Redox control and oxidative stress**								
At3g14950	F4IXE4	TTL2_ARATH TPR repeat-containing thioredoxin TTL2	n.d.	Unique in *pldα1-2*	n.a.	n.a.	6.92	2	6.44	2
At4g34120	Q9C5D0	CBSX2_ARATH CBS domain-containing protein CBSX2	n.d.	0.3	0.085	0.017	6.67	2	12.61	4
At1g65980	Q9XEX2	PRX2B_ARATH Peroxiredoxin-2B	0.53	n.d.	0.048	0.309	10.9	3	29.63	9
At1g76080	Q9SGS4	CDSP_ARATH Thioredoxin-like protein CDSP32	2.36	1.01	0.008	0.979	8.49	2	9.27	17
At1g07890	Q05431	APX1_ARATH L-ascorbate peroxidase 1, cytosolic	1.45	0.77	0.051	0.286	33.85	8	45.6	59
		**Cell wall**								
At5g59290	Q9FIE8	UXS3_ARATH UDP-glucuronic acid decarboxylase 3	Unique in *pldα1-1*	Unique in *pldα1-2*	n.a.	n.a.	4.12/11.97	1/3	9.36/17.54	2/3
At4g34230	O49482	CADH5_ARATH Cinnamyl alcohol dehydrogenase 5	Unique in *pldα1-1*	n.d.	n.a.	n.a.	11.89	3	18.21	3
At1g11580	Q1JPL7	PME18_ARATH Pectinesterase/pectinesterase inhibitor 18	Unique in Col-0	0.59	0.037	0.045	6.54/10.07	2/3	5.74/9.16	2/3
At5g64570	Q9FLG1	BXL4_ARATH Beta-D-xylosidase 4	4.50	0.58	0.053	0.549	15.37	4	10.71	7
At5g16510	Q9FFD2	RGP5_ARATH Probable UDP-arabinopyranose mutase 5	Unique in *pldα1-1*	n.d.	n.a.	n.a.	6.61	2	8.91	2
At1g15950	Q9S9N9	CCR1_ARATH Cinnamoyl-CoA reductase 1	Unique in *pldα1-1*	n.d.	n.a.	n.a.	7.3	2	12.21	2
At4g37800	Q8LER3	XTH7_ARATH Probable xyloglucan endotransglucosylase/hydrolase protein 7	1.45	n.d..	0.001	n.a.	14.95	4	24.23	8
At3g29320	Q9LIB2	PHS1_ARATH Alpha-glucan phosphorylase 1	0.64	0.33	0.202	0.011	29.09	8	15.49	17
		**RNA metabolism, translation**								
At2g04842	F4IFC5	SYTM2_ARATH Threonine--tRNA ligase, chloroplastic/mitochondrial 2	Unique in *pldα1-1*	Unique in *pldα1-2*	n.a.	n.a.	10.45/7.01	3/2	7.08/5.07	3/2
At3g48110	Q8L785	SYGM2_ARATH Glycine--tRNA ligase, chloroplastic/mitochondrial 2	0.429	Unique in Col-0	0.155	n.a.	7.99	2	3.75	2
At4g13780	Q9SVN5	SYMC_ARATH Methionine--tRNA ligase	n.d.	Unique in *pldα1-2*	n.a.	n.a.	7.49	2	4.14	3

At2g33430	O22793	MORF2_ARATH Multiple organellar RNA editing factor 2	0.3	0.44	0.01	0.007	7.9/7.9	2/2	16.44/16.44	8/8
At5g26742	Q8L7S8	RH3_ARATH DEAD-box ATP-dependent RNA helicase 3	0.41	0.58	0.015	0.079	24.38	7	10.56	19
At3g53110	Q93ZG7	RH38_ARATH DEAD-box ATP-dependent RNA helicase 38	1.00	0.18	0.97	0.009	7.54	2	7.06	4
At2g42520	Q84W89	RH37_ARATH DEAD-box ATP-dependent RNA helicase 37	n.d.	Unique in *pldα1-2*	n.a.	n.a.	6.5	2	4.27	2
At4g19210	Q8LPJ4	AB2E_ARATH ABC transporter E family member 2	Unique in *pldα1-1*	n.d.	n.a.	n.a.	10.03	3	10.74	3
At3g05060	Q9MAB3	NOP5B_ARATH Probable nucleolar protein 5-2	Unique in Col-0	1.72	n.a.	0.188	7.37	2	6.75	4
At3g53460	Q43349	CP29A_ARATH 29 kDa ribonucleoprotein	0.49	0.71	0.036	0.349	12.38	3	15.2	15
At5g50250	Q9FGS0	CP31B_ARATH RNA-binding protein CP31B	0.54	n.d.	0.025	n.a.	7.38	2	10.03	9
At5g47210	Q9LVT8	RGGC_ARATH RGG repeats nuclear RNA binding protein C	1.00	0.42	0.991	0.034	14.47	4	19.05	17
At3g25920	P25873	RK15_ARATH 50S ribosomal protein L15	0.62	1.21	0.016	n.a.	24.55	6	25.99	31
At1g05190	O23049	RK6_ARATH 50S ribosomal protein L6	0.29	1.29	0.037	n.a.	14.5	4	25.11	12
AtCg01120	P56805	RR15_ARATH 30S ribosomal protein S15, chloroplastic	0.73	0.59	0.48	0.038	7.4	2	30.68	8
Atcg00160	P56797	RR2_ARATH 30S ribosomal protein S2	n.e.v.	Unique in *pldα1-2*	n.a.	n.a.	10.32	3	21.61	4
At5g56710	P51420	RL313_ARATH 60S ribosomal protein L31-3	n.d.	Unique in *pldα1-2*	n.a.	n.a.	7.81	2	25.21	2
At3g05560	Q9M9W1	RL222_ARATH 60S ribosomal protein L22-2	0.65	0.84	0.042	0.175	13.89	4	41.13	12
At3g62870	Q9LZH9	RL7A2_ARATH 60S ribosomal protein L7a-2	1.35	2.50	0.458	0.041	11.16	3	14.06	8
At3g24830	Q9LRX8	R13A2_ARATH 60S ribosomal protein L13a-2	n.e.v.	1.73	n.a.	0.038	7.32	2	12.62	3
At4g31700	O48549	RS61_ARATH 40S ribosomal protein S6-1	0.22	0.78	0.001	0.423	12.06	3	16.4	14
At4g39200	Q9T029	RS254_ARATH 40S ribosomal protein S25-4	0.93	0.49	0.801	0.036	15.33	4	26.85	13
At3g45030	P49200	RS201_ARATH 40S ribosomal protein S20-1	0.99	0.30	0.964	0.023	10.56	3	29.03	10
At5g10360	P51430	RS62_ARATH 40S ribosomal protein S6-2	0.56	0.93	0.004	0.700	16.59	4	23.69	32
At2g36160	Q9SIH0	RS141_ARATH 40S ribosomal protein S14-1	1.91	0.55	0.03	0.154	17.63	4	30	15

At3g46040	Q9LX88	R15A4_ARATH 40S ribosomal protein S15a-4	8.51	1.16	0.02	0.877	8.62	2	26.15	12
At1g48830	Q9C514	RS71_ARATH 40S ribosomal protein S7-1	0.28	0.18	0.214	0.007	18.15	4	30.89	10
At4g34555	Q8GYL5	RS253_ARATH 40S ribosomal protein S25-3	0.79	0.58	0.311	0.041	11.76	3	25	17
At1g36730	Q9C8F1	IF5Y_ARATH Probable eukaryotic translation initiation factor 5-1	Unique in *pldα1-1*	n.d.	n.a.	n.a.	6.2	2	6.15	2
At2g39990	O04202	EIF3F_ARATH Eukaryotic translation initiation factor 3 subunit F	Unique in Col-0	0.73	n.a.	0.165	8.74	2	12.29	4
At1g10840	Q9C5Z2	EIF3H_ARATH Eukaryotic translation initiation factor 3 subunit H	1.09	0.69	0.741	0.004	10.39	3	12.76	3
At3g63190	Q9M1X0	PG_ARATH 4-hydroxy-3-methylbut-2-en-1-yl diphosphate synthase (ferredoxin)	n.a.	0.54	n.a.	0.049	18.65	4	21.82	29
At3g49470	Q94JX9	NACA2_ARATH Nascent polypeptide-associated complex subunit alpha-like protein 2	0.61	0.63	0.384	0.041	13.99	3	19.82	14
At4g27000	Q93W34	RP45C_ARATH Polyadenylate-binding protein RBP45C	1.20	Unique in Col-0	0.788	n.a.	7.67	2	9.4	4
At1g02150	Q8LPS6	PPR3_ARATH Pentatricopeptide repeat-containing protein At1g02150	1.53	Unique in Col-0	0.321	n.a.	6.57	2	6.11	2
At2g24060	O82234	IF32_ARATH Translation initiation factor IF3-2	2.04	1.69	0.022	0.188	6.42	2	8.97	3
At3g57150	Q9LD90	CBF5_ARATH H/ACA ribonucleoprotein complex subunit 4	1.69	n.d.	0.031	n.a.	9.95	2	3.19	6
At5g02490	P22954	MD37D_ARATH Probable mediator of RNA polymerase II transcription subunit 37c	0.69	0.65	0.141	0.02	61.15	14	30.47	95
At3g51800	Q96327	EBP1_ARATH ERBB-3 BINDING PROTEIN 1	0.33	1.56	0.200	0.044	19.37	5	15.05	12
At3g12580	Q9LHA8	MD37C_ARATH Probable mediator of RNA polymerase II transcription subunit 37c	0.86	0.64	0.272	0.027	75	18	35.85	103
		**Secondary metabolism**								
At5g54160	Q9FK25	OMT1_ARATH Flavone 3'-O-methyltransferase 1	1.83	0.99	0.001	0.977	31.46	8	30.85	41
At5g23010	Q9FG67	MAM1_ARATH Methylthioalkylmalate synthase 1, chloroplastic	Unique in Col-0	0.66	n.a.	0.168	11.19	3	10.87	5
At2g43100	Q9ZW84	LEUD1_ARATH 3-isopropylmalate dehydratase small subunit 1	0.45	0.58	0.001	0.002	11.25/11.43	3/3	19.92/19.92	4/6

At3g61220	Q9M2E2	SDR1_ARATH (+)-neomenthol dehydrogenase	1.95	2.89	0.022	0.026	16.50/22.76	4/6	26.69/30.74	11/20
At3g02780	Q42553	IDI2_ARATH Isopentenyl-diphosphate Delta-isomerase II, chloroplastic	3.27	2.55	0.053	0.446	8.63	2	12.32	6
At5g60600	F4K0E8	ISPG_ARATH 4-hydroxy-3-methylbut-2-en-1-yl diphosphate synthase (ferredoxin), chloroplastic	2.58	1.95	0.039	0.178	29.49	8	17.68	25
At1g24100	O48676	U74B1_ARATH UDP-glycosyltransferase 74B1	Unique in Col-0	0.49	n.a.	0.352	6.81	2	5.87	3
At4g14210	Q07356	PDS_ARATH 15-cis-phytoene desaturase, chloroplastic/chromoplastic	n.d.	Unique in *pldα1-2*	n.a.	n.a.	10.78	3	8.3	3
At2g29290	Q9ZW13	TRNH6_ARATH Tropinone reductase homolog	n.e.v.	Unique in *pldα1-2*	n.a.	n.a.	7.21	2	10.69	3
		**Transport, Membrane transport, transmembrane transport**								
At5g16620	Q9FMD5	TIC40_ARATH Protein TIC 40, chloroplastic	0.61	0.28	0.152	0.61	24.2	6	24.16	11
At1g76030	P11574	VATB1_ARATH V-type proton ATPase subunit B1	0.59	0.74	0.009	0.59	58.85	13	47.12	123
At5g58440	Q8L5Z7	SNX2A_ARATH Sorting nexin 2A	1.40	Unique in Col-0	0.459	1.40	6.55	2	4.43	4
		**Protein/peptide degradation**								
At5g43060	Q9FMH8	RD21B_ARATH Probable cysteine protease RD21B	n.d.	Unique in *pldα1-2*	n.a.	n.a.	6.76	2	6.05	2
At1g49970	Q9XJ35	CLPR1_ARATH ATP-dependent Clp protease proteolytic subunit-related protein 1, chloroplastic	Unique in Col-0	0.28	n.a.	0.156	11.09	3	13.18	5
At1g09130	Q8L770	CLPR3_ARATH ATP-dependent Clp protease proteolytic subunit-related protein 3, chloroplastic	4.92	Unique in Col-0	0.243	n.a.	8.61	2	12.12	2
At4g25370	Q93WL3	CLPT1_ARATH ATP-dependent Clp protease ATP-binding subunit CLPT1, chloroplastic	n.e.v.	Unique in *pldα1-2*	n.a.	n.a.	7.61	2	18.07	3
At1g13060	O23717	PSB5A_ARATH Proteasome subunit beta type-5-A	0.43	0.52	0.032	0.235	15.05	4	30.29	8
At5g42190	Q9FHW7	SKP1B_ARATH SKP1-like protein 1B	Unique in *pldα1-1*	Unique in *pldα1-2*	n.a.	n.a.	8.49/8.67	2/2	19.88/19.88	4/3
At1g75950	Q39255	SKP1A_SKP1-like protein 1A	Unique in Col-0	0.54	n.a.	0.412	12.04	3	30.63	10
At5g09900	Q9FIB6	PS12A_ARATH 26S proteasome non-ATPase regulatory subunit 12 homolog A	Unique in Col-0	0.47	n.a.	0.28	7.53	2	8.82	2

At2g47940	O82261	DEGP2_ARATH Protease Do-like 2, chloroplastic	Unique in Col-0	0.82	n.a.	0.597	6.83	2	6.43	4
At4g11260	Q9SUT5	SGT1B_ARATH Protein SGT1 homolog B	n.d.	Unique in *pldα1-2*	n.a.	n.a.	10.58	3	12.29	4
At1g45000	Q9MAK9	PS10B_ARATH 26S proteasome regulatory subunit S10B homolog B	0.74	2.30	0.008	0.116	7	2	8.02	3
		**Photosynthetic and respiratory electron flow**								
At3g52300	Q9FT52	ATP5H_ARATH ATP synthase subunit d, mitochondrial	0.37	0.69	0.06	0.032	7.48	2	15.48	8
Atcg00130	P56759	ATPF_ARATH ATP synthase subunit b, chloroplastic	Unique in *pldα1-1*	3.78	n.a.	0.001	7.04/12.00	2/3	18.48/28.80	3/9
At1g44575	Q9XF91	PSBS_ARATH Photosystem II 22 kDa protein, chloroplastic	0.43	0.24	0.011	0.27	16.57	4	13.58	18
At3g56650	Q9LXX5	PPD6_ARATH PsbP domain-containing protein 6, chloroplastic	0.8	0.27	0.581	0.029	8.27	2	14.12	9
At1g03600	Q9LR64	PB27A_ARATH Photosystem II repair protein PSB27-H1, chloroplastic	0.57	0.67	0.09	0.045	16.53	4	28.16	34
At5g53490	P81760	TL17_ARATH Thylakoid lumenal 17.4 kDa protein, chloroplastic	2.19	2.35	0.005	0.095	8.69	2	10.59	12
At3g61470	Q9SYW8	LHCA2_ARATH Photosystem I chlorophyll a/b-binding protein 2, chloroplastic	1.14	6.23	0.815	0.007	15.66	4	24.9	13
At1g61520	Q9SY97	LHCA3_ARATH Photosystem I chlorophyll a/b-binding protein 3-1, chloroplastic	0.9	2.1	0.65	0.049	31.18	7	41.03	54
At4g17600	Q9SYX1	LIL31_ARATH Light-harvesting complex-like protein 3 isotype 1, chloroplastic	n.d.	Unique in *pldα1-2*	n.a.	n.a.	6.9	2	14.89	3
At4g21280	Q9XFT3-2	PSBQ1_ARATH Isoform 2 of Oxygen-evolving enhancer protein 3-1, chloroplastic	0.58	0.53	0.008	0.017	38.19/30.88	9/7	46.64/43.50	100/96
At4g03280	Q9ZR03	UCRIA_ARATH Cytochrome b6-f complex iron-sulfur subunit, chloroplastic	0.92	1.69	0.738	0.03	25.75	6	41.05	43
At4g22890	Q8H112	PL1A_ARATH PR5-like protein 1A, chloroplastic	6.28	5.54	0.001	0.001	11.71/15.29	3/4	14.81/14.81	9/9
		**Metabolism**								
At3g13750	Q9SCW1	BGAL1_ARATH Beta-galactosidase 1	n.e.v.	Unique in *pldα1-2*	n.a.	n.a.	13.9	4	8.74	4
At4g38800	Q9T0I8	MTN1_ARATH 5'-methylthioadenosine/S-adenosylhomocysteine nucleosidase 1	0.49	Unique in Col-0	0.073	n.a.	9.94	2	17.98	4

At4g39980	P29976	AROF_ARATH Phospho-2-dehydro-3-deoxyheptonate aldolase 1, chloroplastic	n.d.	Unique in *pldα1-2*	n.a.	n.a.	9.89	3	9.33	3
At4g21150	Q93Z16	RPN2_ARATH Dolichyl-diphosphooligosaccharide--protein glycosyltransferase subunit 2	1.08	Unique in Col-0	0.737	n.a.	7.43	2	4.63	3
At5g11950	Q84MC2	LOG8_ARATH Cytokinin riboside 5'-monophosphate phosphoribohydrolase LO	n.d.	Unique in *pldα1-2*	n.a.	n.a.	6.97	2	22.69	2
At5g37510	Q9FGI6	NDUS1_ARATH NADH dehydrogenase [ubiquinone] iron-sulfur protein 1, mitochondrial	Unique in Col-0	0.72	n.a.	0.491	7	2	4.01	3
At5g22300	P46011	NRL4_ARATH Bifunctional nitrilase/nitrile hydratase NIT4	Unique in *pldα1-1*	n.d.	n.a.	n.a.	6.83	2	8.45	4
At1g09795	Q8GSJ1	HIS1B_ARATH ATP phosphoribosyltransferase 2, chloroplastic	Unique in *pldα1-1*	n.e.v.	n.a.	n.a.	6.66	2	8.23	2
At4g08900	P46637	ARGI1_ARATH Arginase 1, mitochondrial	Unique in *pldα1-1*	n.e.v.	n.a.	n.a.	6.44	2	7.02	2
At2g31810	Q93YZ7	ILVH2_ARATH Acetolactate synthase small subunit 2, chloroplastic	Unique in *pldα1-1*	n.d.	n.a.	n.a.	6.41	2	5.91	2
At3g54110	O81845	PUMP1_ARATH Mitochondrial uncoupling protein 1	Unique in Col-0	1.51	n.a.	0.767	6.4	2	11.44	2
At3g01120	P55217	CGS1_ARATH Cystathionine gamma-synthase 1, chloroplastic	Unique in *pldα1-1*	Unique in *pldα1-2*	n.a.	n.a.	6.36/7.77	2/2	7.82/6.93	2/2
At1g31860	O82768	HIS2_ARATH Histidine biosynthesis bifunctional protein hisIE, chloroplastic	Unique in *pldα1-1*	n.e.v.	n.a.	n.a.	7.23	2	11.74	2
At4g36810	P34802	GGPP1_ARATH Heterodimeric geranylgeranyl pyrophosphate synthase large subunit 1, chloroplastic	Unique in *pldα1-1*	n.e.v.	n.a.	n.a.	8.25	2	14.02	4
At5g13490	P40941	ADT2_ARATH ADP,ATP carrier protein 2, mitochondrial	Unique in Col-0	Unique in Col-0	n.a.	n.a.	8.21/8.21	2/2	8.31/8.31	2/2
At5g55070	Q9FLQ4	ODO2A_ARATH Dihydrolipoyllysine-residue succinyltransferase component of 2-oxoglutarate dehydrogenase complex 1, mitochondrial	Unique in Col-0	0.39	n.a.	0.104	8.21	2	8.62	5
At4g37870	Q9T074	PCKA_ARATH Phosphoenolpyruvate carboxykinase (ATP)	Unique in *pldα1-1*	n.d.	n.a.	n.a.	10.01	3	7	3
At5g10920	Q9LEU8	ARLY_ARATH Argininosuccinate lyase, chloroplastic	Unique in *pldα1-1*	Unique in *pldα1-2*	n.a.	n.a.	11.08/3.69	3/1	12.19/3.87	4
At5g18170	Q43314	DHE1_ARATH Glutamate dehydrogenase 1	0.55	1.03	0.005	0.904	11.18	3	11.44	6

At3g59760	Q43725	CYSKM_ARATH Cysteine synthase, mitochondrial	0.26	0.93	0.007	0.924	12.95	4	15.12	4
At5g01410	Q8L940	PDX13_ARATH Pyridoxal 5'-phosphate synthase subunit PDX1.3	1.49	1.85	0.053	0.018	23.65/23.94	6/6	29.77/29.77	28/28
At4g38970	Q944G9	ALFP2_ARATH Fructose-bisphosphate aldolase 2, chloroplastic	0.78	0.87	0.013	0.188	67.7	15	41.96	165
At1g06690	Q94A68	Y1669_ARATH Uncharacterized oxidoreductase At1g06690, chloroplastic	0.11	0.71	0.02	0.564	9.94	3	14.85	3
At4g35630	Q96255	SERB1_ARATH Phosphoserine aminotransferase 1, chloroplastic	0.8	0.41	0.662	0.003	17.63	4	14.65	23
At1g32220	Q9FVR6	Y1222_ARATH Uncharacterized protein At1g32220, chloroplastic	1.25	3.05	0.774	0.031	23.77	6	35.14	12
At3g19480	Q9LT69	SERA3_ARATH D-3-phosphoglycerate dehydrogenase 3, chloroplastic	0.53	0.65	0.459	0.001	6.35	2	4.93	3
At4g31990	P46248	AAT5_ARATH Aspartate aminotransferase, chloroplastic	1.97	1.27	0.051	0.52	28.63	8	25.83	21
At3g61440	Q9S757	CYSC1_ARATH Bifunctional L-3-cyanoalanine synthase/cysteine synthase C1, mitochondrial	2.26	1.65	0.108	0.034	15.5	4	11.41	22
At2g44350	P20115	CISY4_ARATH Citrate synthase 4, mitochondrial	3.53	2.03	0.001	0.311	12.05	3	11.18	8
At4g01900	Q9ZST4	GLNB_ARATH Nitrogen regulatory protein P-II homolog	0.46	0.49	0.003	0.325	11.09	3	13.78	7
At5g51820	Q9SCY0	PGMP_ARATH Phosphoglucomutase, chloroplastic	0.55	0.59	0.035	0.092	38.74	10	23.92	38
At2g31390	Q9SID0	SCRK1_ARATH Probable fructokinase-1	Unique in Col-0	0.4	n.a.	0.209	14.62	4	21.85	6
At1g23740	Q9ZUC1	AOR_ARATH NADPH-dependent alkenal/one oxidoreductase, chloroplastic	0.49	0.98	0.022	0.895	45.4	12	50	48
At1g12240	Q39041	INVA4_ARATH Acid beta-fructofuranosidase 4, vacuolar	1.87	0.68	0.009	0.31	14.06	4	9.94	11
At5g11880	Q94A94	DCDA2_ARATH Diaminopimelate decarboxylase 2, chloroplastic	0.5	0.68	0.042	0.308	7	2	5.52	4
At5g08530	Q9FNN5	NDUV1_ARATH NADH dehydrogenase [ubiquinone] flavoprotein 1, mitochondrial	0.38	0.44	0.001	0.296	6.98	2	5.76	3
At5g43780	Q9S7D8	APS4_ARATH ATP sulfurylase 4, chloroplastic	0.82	0.11	0.648	0.025	10.67	3	8.74	7
At4g20960	Q8GWP5	RIBD_ARATH Riboflavin biosynthesis protein PYRD, chloroplastic	0.57	0.37	0.381	0.032	10.17	3	12.44	5

At5g47840	Q9FIJ7	KAD2_ARATH Adenylate kinase 2, chloroplastic	Unique in *pldα1-1*	Unique in *pldα1-2*	n.a.	n.a.	11.01/8.51	3/2	19.43/14.49	3/4
At1g31812	P57752	ACBP6_ARATH Acyl-CoA-binding domain-containing protein 6	n.d.	Unique in *pldα1-2*	n.a.	n.a.	6.82	2	35.87	2
At5g64300	P47924	RIBA1_ARATH Bifunctional riboflavin biosynthesis protein RIBA 1, chloroplastic	n.d.	Unique in *pldα1-2*	n.a.	n.a.	6.6	2	6.45	2
At4g23890	Q9T0A4	NDHS_ARATH NAD(P)H-quinone oxidoreductase subunit S, chloroplastic	1.09	Unique in Col-0	0.8	n.a.	7.17	2	11.6	3
At4g39970	Q680K2	GPPL1_ARATH Haloacid dehalogenase-like hydrolase domain-containing protein At4g39970	n.d.	Unique in *pldα1-2*	n.a.	n.a.	7.17	2	9.18	4
At2g13560	Q9SIU0	MAO1_ARATH NAD-dependent malic enzyme 1, mitochondrial	0.52	Unique in Col-0	0.471	n.a.	7.04	2	6.26	2
At5g59420	Q93Y40	ORP3C_ARATH Oxysterol-binding protein-related protein 3C	n.e.v.	Unique in *pldα1-2*	n.a.	n.a.	8.23	2	6.78	3
At4g19710	O81852	AKH2_ARATH Bifunctional aspartokinase/homoserine dehydrogenase 2, chloroplastic	0.55	Unique in Col-0	0.241	n.a.	8.17	2	4.26	3
At3g23940	Q9LIR4	ILVD_ARATH Dihydroxy-acid dehydratase, chloroplastic	Unique in Col-0	0.31	n.a.	0.04	7.31/7.59	2/2	6.09/6.09	3/5
At4g26970	Q94A28	ACO2M_ARATH Aconitate hydratase 2, mitochondrial	3.16	3.27	0.17	0.054	28.65	7	11.16	17
At1g70730	Q9SGC1	PGMC2_ARATH Probable phosphoglucomutase, cytoplasmic 2	0.93	0.68	0.849	0.023	42.08	11	33.68	30
At1g62640	P49243	FABH_ARATH 3-oxoacyl-[acyl-carrier-protein] synthase III, chloroplastic	0.93	0.66	0.847	0.01	7.5	2	7.92	5
At3g16000	Q9LW85	MFP1_ARATH MAR-binding filament-like protein 1	1.05	0.51	0.915	0.025	7.02	2	4.13	3
At5g66120	Q8VYV7	DHQS_ARATH 3-dehydroquinate synthase, chloroplastic	0.62	0.41	0.397	0.036	8.15	2	8.82	5
At3g57610	Q96529	PURA_ARATH Adenylosuccinate synthetase, chloroplastic	0.76	0.39	0.121	0.023	16.75	4	15.1	14
At3g06650	Q9C522	ACLB1_ARATH ATP-citrate synthase beta chain protein 1	1.77	4.06	0.365	0.007	11.18	3	8.06	6
At3g23490	O22683	CYNS_ARATH Cyanate hydratase OS=Arabidopsis thaliana GN=CYN PE=2 SV=1	1.515	3.53	0.565	0.047	9.11	2	26.19	7
At3g13930	Q8RWN9	ODP22_ARATH Dihydrolipoyllysine-residue acetyltransferase component 2 of pyruvate dehydrogenase complex, mitochondrial	0.98	3.32	0.977	0.026	6.8	2	4.64	3
At3g20050	P28769	TCPA_ARATH T-complex protein 1 subunit alpha	n.e.v.	1.84	n.a.	0.006	11.87	3	7.34	8

At4g26530	O65581	ALFC5_ARATH Fructose-bisphosphate aldolase 5, cytosolic	1.26	1.66	0.086	0.025	55.23	12	58.38	81
At2g38230	O80448	PDX11_ARATH Pyridoxal 5'-phosphate synthase subunit PDX1.1	1.27	1.66	0.24	0.038	29.44	7	25.24	43
At1g43670	Q9MA79	F16P2_ARATH Fructose-1,6-bisphosphatase, cytosolic	1.24	1.65	0.24	0.039	36.13	8	41.35	46
At2g33150	Q56WD9	THIK2_ARATH 3-ketoacyl-CoA thiolase 2, peroxisomal	0.84	0.63	0.426	0.055	35.58	8	30.09	44
At5G11670	Q9LYG3	MAOP2_ARATH NADP-dependent malic enzyme 2	1.07	0.55	0.595	0.008	41.28	11	33.5	27
At1g78570	Q9SYM5	RHM1_ARATH Trifunctional UDP-glucose 4,6-dehydratase/UDP-4-keto-6-deoxy-D-glucose 3,5-epimerase/UDP-4-keto-L-rhamnose-reductase RHM1	Unique in Col-0	Unique in Col-0	n.a.	n.a.	7.71/7.71	2/2	3.44/3.44	3/3
At1g31230	Q9SA18	AKH1_ARATH Bifunctional aspartokinase/homoserine dehydrogenase 1, chloroplastic	0.75	0.11	0.585	0.013	7.95	2	4.06	3
		**Stress response**								
At5g02500	P22953	MD37E_ARATH Probable mediator of RNA polymerase II transcription subunit 37e	0.73	0.5	0.031	0.003	137.76/127.79	32/29	57.91/57.91	226/209
At4g16260	Q8VZJ2	BGNEM_ARATH Probable glucan endo-1,3-beta-glucosidase At4g16260	n.e.v.	Unique in *pldα1-2*	n.a.	n.a.	10.2	3	17.73	4
At3g15356	Q9LJR2	LECT2_ARATH Lectin-like protein LEC	4.23	1.3	0.021	0.804	19.58	5	29.89	18
At1g78370	Q8L7C9	GSTUK_ARATH Glutathione S-transferase U20	1.12	1.69	0.859	0.046	7.6	2	11.06	11
At4g33510	Q00218	AROG_ARATH Phospho-2-dehydro-3-deoxyheptonate aldolase 2, chloroplastic	2.01	n.e.v.	0.026	n.a.	11.36	3	12.03	8
At2g29450	P46421	GSTU5_ARATH Glutathione S-transferase U5	3.07	1.57	0.004	0.383	14.65	4	25	9
At5g16710	Q8LE52	DHAR3_ARATH Glutathione S-transferase DHAR3, chloroplastic	0.24	0.66	0.042	0.361	8.9	2	12.79	11
At5g22580	Q9FK81	Y5258_ARATH Stress-response A/B barrel domain-containing protein At5g22580	0.58	0.32	0.038	0.001	7.14/7.28	2/2	22.52/22.52	5/5
At5g65020	Q9XEE2	ANXD2_ARATH Annexin D2	Unique in *pldα1-1*	Unique in *pldα1-2*	n.a.	n.a.	3.51/6.49	1/2	5.99/10.09	2/2
At1g53280	Q9MAH3	DJ1B_ARATH Protein DJ-1 homolog B	1.86	Unique in Col-0	0.2	n.a.	7.38	2	9.59	4
At3g15730	Q38882	PLDA1_ARATH Phospholipase D alpha 1	Unique in Col-0	Unique in Col-0	n.a.	n.a.	12.06/12.06	3/3	8.15/8.15	8/8

At1g78380	Q9ZRW8	GSTUJ_ARATH Glutathione S-transferase U19	0.78	0.51	0.527	0.053	7.08	2	12.79	13
At5g64120	Q43387	PER71_ARATH Peroxidase 71	0.78	0.5	0.403	0.033	10.76	3	17.07	8
At4g09320	P39207	NDK1_ARATH Nucleoside diphosphate kinase 1	0.56	0.63	0.154	0.054	29.55	7	55.03	37
At4g08390	Q42592	APXS_ARATH L-ascorbate peroxidase S, chloroplastic/mitochondrial	1.41	0.38	0.528	0.027	27.21	7	33.33	19
At3g28940	Q9MBH2	AIG2B_ARATH Protein AIG2B	0.68	0.61	0.045	0.218	7.77	2	23.08	10
		**Protein folding**								
At3g13860	Q93ZM7	CH60C_ARATH Chaperonin CPN60-like 2, mitochondrial	Unique in *pldα1-1*	Unique in *pldα1-2*	n.a.	n.a.	6.24/3.49	2/1	6.12/3.5	2/2
At4g34870	Q42406	CP18D_ARATH Peptidyl-prolyl cis-trans isomerase CYP18-4	0.52	0.48	0.042	0.03	20.24/20.56	4/4	34.30/34.30	33/35
At5g13410	Q9LYR5	FKB19_ARATH Peptidyl-prolyl cis-trans isomerase FKBP19, chloroplastic	n.e.v.	1.65	n.a.	0.039	10.46	3	21.48	4
At2g43560	O22870	FK163_ARATH Peptidyl-prolyl cis-trans isomerase FKBP16-3, chloroplastic	0.69	0.6	0.168	0.019	8.1	2	11.21	6
At3g09440	O65719	HSP7C_ARATH Heat shock 70 kDa protein 3	0.79	0.72	0.151	0.052	106.7	26	55.16	146
At5g60640	Q9FF55	PDI14_ARATH Protein disulfide isomerase-like 1-4	Unique in Col-0	3.84	n.a.	0.006	4.1/7.3	1/2	2.85/5.19	3/8
At2g47470	O22263	PDI21_ARATH Protein disulfide-isomerase like 2-1	0.47	0.31	0.042	0.051	14.89/14.89	4/4	13.57/14	14/14
At5g52640	P27323	HS901_ARATH Heat shock protein 90-1	0.79	0.58	0.459	0.002	10.2	3	6.43	12
At1g12270	Q9LNB6	HSOP1_ARATH Hsp70-Hsp90 organizing protein 1	0.96	Unique in Col-0	0.947	n.a.	8.54	2	8.04	3
At3g44110	Q94AW8	DNAJ3_ARATH Chaperone protein dnaJ 3	0.43	1.11	0.033	0.764	14.63	4	16.67	9
At3g23990	P29197	CH60A_ARATH Chaperonin CPN60, mitochondrial	0.63	0.64	0.052	0.175	46	11	32.93	40
At5g50920	Q9FI56	CLPC1_ARATH Chaperone protein ClpC1, chloroplastic	0.8	0.91	0.052	0.553	116.42	28	41.44	149
At3g48870	Q9SXJ7	CLPC2_ARATH Chaperone protein ClpC2, chloroplastic	0.77	0.92	0.033	0.56	111.6	27	39.08	130
At1g16030	Q9S9N1	HSP7E_ARATH Heat shock 70 kDa protein 5	0.44	0.63	0.023	0.095	20.64	5	11.46	34
At3g03960	Q94K05	TCPQ_ARATH T-complex protein 1 subunit theta	0.74	0.5	0.396	0.05	10.76	3	8.56	5
At5g15450	Q9LF37	CLPB3_ARATH Chaperone protein ClpB3, chloroplastic	0.35	0.41	0.051	0.103	22.85	6	10.54	11

		**Cell division**								
At1g05560	Q9LR44	U75B1_ARATH UDP-glycosyltransferase 75B1	0.74	0.27	0.57	0.026	7.44	2	6.18	4
At1g72150	Q56WK6	PATL1_ARATH Patellin-1	1.04	4.44	0.952	0.045	33.7	9	21.82	24
At1g47200	Q9C500	WPP2_ARATH WPP domain-containing protein 2	0.77	7.21	0.225	0.025	6.98	2	19.44	3
		**Chlorophyll biosynthesis**								
At4g18480	P16127	CHLI1_ARATH Magnesium-chelatase subunit ChlI-1, chloroplastic	0.53	0.52	0.046	0.021	41.47/44.51	10/11	36.56/39.62	50/54
At3g56940	Q9M591	CRD1_ARATH Magnesium-protoporphyrin IX monomethyl ester [oxidative] cyclase, chloroplastic	0.44	0.47	0.037	0.31	14.42	4	13.94	14
At5g08280	Q43316	HEM3_ARATH Porphobilinogen deaminase, chloroplastic	0.41	0.48	0.05	0.104	27.07	7	27.23	26
At4g25080	Q9SW18	CHLM_ARATH Magnesium protoporphyrin IX methyltransferase, chloroplastic	1.01	5.58	0.937	0.023	7.2	2	9.29	5
		**Chloroplast biogenesis**								
At5g19620	Q9C5J8	OEP80_ARATH Outer envelope protein 80, chloroplastic	Unique in Col-0	1.92	n.a.	0.102	6.97	2	4.92	2
		**Circadian clock**								
At3g22380	Q94KE2	TIC_ARATH Protein TIME FOR COFFEE	Unique in Col-0	1.49	n.a.	0.07	7.05	2	2.52	2
		**Signalling**								
At1g08450	O04153	CALR3_ARATH Calreticulin-3	Unique in Col-0	0.35	n.a.	0.053	6.73/6.73	2/2	7.55/7.55	2/3
		**Cytoskeleton**								
At1g18450	Q84M92	ARP4_ARATH Actin-related protein 4	n.d.	Unique in *pldα1-2*	n.a.	n.a.	6.72	2	8.84	2
At5g59880	Q9ZSK4	ADF3_ARATH Actin-depolymerizing factor 3	0.95	0.62	0.862	0.05	14.54	3	43.17	15
At3g57410	O81645	VILI3_ARATH Villin-3	0.88	0.34	0.833	0.01	14.29	4	6.94	6
		**Storage protein**								
At4g28520	Q96318	CRU3_ARATH 12S seed storage protein CRC	n.d.	Unique in *pldα1-2*	n.a.	n.a.	8.58	2	7.82	2
		**Unknown**								
At4g15545	Q93W28	Y4554_ARATH Uncharacterized protein At4g15545	n.e.v.	Unique in *pldα1-2*	n.a.	n.a.	7.01	2	7.12	2
At1g55590	Q9ZWC6	ATB_ARATH F-box protein At-B	n.e.v.	Unique in *pldα1-2*	n.a.	n.a.	6.56	2	4.78	2

At1g48610	Q94AD1	D1861_ARATH Putative DNA-binding protein At1g48610	n.d.	Unique in *pldα1-2*	n.a.	n.a.	6.51	2	16.51	2
At4g13200	Q8LDV3	Y4320_ARATH Uncharacterized protein At4g13200, chloroplastic	Unique in Col-0	0.31	n.a.		3.43/6.67	1/2	7.57/17.3	3/4

Table S2. List of proteins differentially abundant in above ground parts of *pldα1-1* and *pldα1-2* mutants predicted by ChloroP server to contain chloroplast transit peptides (cTP) in their aminoacid sequence. CS = cleavage site

Accession			fold change		*p* value		predicition of chloroplast transition sequence (ChloroP)				
TAIR	UNIPROT	Sequence Name	*pldα1-1* vs Col-0	*pldα1-2* vs Col-0	*pldα1-1* vs Col-0	*pldα1-2* vs Col-0	Length	Score	cTP	CS-score	cTP-length
		**RNA metabolism, translation**									
At5g50250	Q9FGS0	CP31B_ARATH RNA-binding protein CP31B	0.54	n.d.	0.025	n.a.	283	0.541	Y	11.31	57
At2g42520	Q84W89	RH37_ARATH DEAD-box ATP-dependent RNA helicase 37	n.d.	Unique in *pldα1-2*	n.a.	n.a.	588	0.567	Y	3.531	38
At3g53460	Q43349	CP29A_ARATH 29 kDa ribonucleoprotein	0.49	0.71	0.036	0.349	557	0.533	Y	0.638	42
At3g25920	P25873	RK15_ARATH 50S ribosomal protein L15	0.62	1.21	0.016	0.464	196	0.525	Y	5.228	61
At2g24060	O82234	IF32_ARATH Translation initiation factor IF3-2	2.04	1.69	0.022	0.188	424	0.589	Y	6.703	60
At1g05190	O23049	RK6_ARATH 50S ribosomal protein L6	0.29	1.29	0.037	0.537	371	0.57	Y	4.926	56
At2g33430	O22793	MORF2_ARATH Multiple organellar RNA editing factor 2	0.3	0.44	0.01	0.007	368	0.501	Y	-1.197	24
At2g04842	F4IFC5	SYTM2_ARATH Threonine--tRNA ligase, chloroplastic/mitochondrial 2	Unique in *pldα1-1*	Unique in *pldα1-2*	n.a.	n.a.	273	0.584	Y	0.751	48
At3g48110	Q8L785	SYGM2_ARATH Glycine--tRNA ligase, chloroplastic/mitochondrial 2	0.429	Unique in Col-0	0.155	n.a.	460	0.516	Y	0.936	34
At5g26742	Q8L7S8	RH3_ARATH DEAD-box ATP-dependent RNA helicase 3	0.41	0.58	0.015	0.079	608	0.549	Y	7.286	35
At4g27000	Q93W34	RP45C_ARATH Polyadenylate-binding protein RBP45C	1.2	Unique in Col-0	0.788	n.a.	312	0.585	Y	9.847	39
At1g02150	Q8LPS6	PPR3_ARATH Pentatricopeptide repeat-containing protein At1g02150	1.53	Unique in Col-0	0.321	n.a.	623	0.564	Y	2.546	52
At2g39990	O04202	EIF3F_ARATH Eukaryotic translation initiation factor 3 subunit F	Unique in Col-0	0.73	n.a.	0.165	324	0.598	Y	4.945	60
		**Proteolysis**									
At4g25370	Q93WL3	CLPT1_ARATH ATP-dependent Clp protease ATP-binding subunit CLPT1, chloroplastic	n.e.v.	Unique in *pldα1-2*	n.a.	n.a.	607	0.584	Y	1.071	69
At1g49970	Q9XJ35	CLPR1_ARATH ATP-dependent Clp protease proteolytic subunit-related protein 1, chloroplastic	Unique in Col-0	0.28	n.a.	0.156	296	0.566	Y	8.074	57
At1g09130	Q8L770	CLPR3_ARATH ATP-dependent Clp protease proteolytic subunit-related protein 3, chloroplastic	4.92	Unique in Col-0	0.243	n.a.	250	0.549	Y	7.705	48
At2g47940	O82261	DEGP2_ARATH Protease Do-like 2, chloroplastic	Unique in Col-0	0.82	n.a.	0.597	262	0.585	Y	9.136	39
		**Chlorophyll biosynthesis**									
At4g18480	P16127	CHLI1_ARATH Magnesium-chelatase subunit ChlI-1, chloroplastic	0.53	0.52	0.046	0.021	563	0.563	Y	8.988	103
At4g25080	Q9SW18	CHLM_ARATH Magnesium protoporphyrin IX methyltransferase, chloroplastic	1.01	5.58	0.937	0.023	415	0.506	Y	-0.001	61

At3g56940	Q9M591	CRD1_ARATH Magnesium-protoporphyrin IX monomethyl ester [oxidative] cyclase, chloroplastic	0.44	0.47	0.037	0.31	277	0.567	Y	4.436	65
		**Photosynthesis**									
At4g22890	Q8H112	PL1A_ARATH PR5-like protein 1A, chloroplastic	6.28	5.54	0.001	0.001	386	0.533	Y	2.101	2
At4g17600	Q9SYX1	LIL31_ARATH Light-harvesting complex-like protein 3 isotype 1, chloroplastic	n.d.	Unique in *pldα1-2*	n.a.	n.a.	236	0.525	Y	6.571	33
At1g61520	Q9SY97	LHCA3_ARATH Photosystem I chlorophyll a/b-binding protein 3-1, chloroplastic	0.9	2.1	0.65	0.049	726	0.592	Y	3.688	41
At3g56650	Q9LXX5	PPD6_ARATH PsbP domain-containing protein 6, chloroplastic	0.8	0.27	0.581	0.029	413	0.597	Y	3.382	57
At1g03600	Q9LR64	PB27A_ARATH Photosystem II repair protein PSB27-H1, chloroplastic	0.57	0.67	0.09	0.045	784	0.546	Y	0.304	45
At4g03280	Q9ZR03	UCRIA_ARATH Cytochrome b6-f complex iron-sulfur subunit, chloroplastic	0.92	1.69	0.738	0.03	185	0.585	Y	3.261	56
At3g61470	Q9SYW8	LHCA2_ARATH Photosystem I chlorophyll a/b-binding protein 2, chloroplastic	1.14	6.23	0.815	0.007	316	0.586	Y	1.674	46
At5g53490	P81760	TL17_ARATH Thylakoid lumenal 17.4 kDa protein, chloroplastic	2.19	2.35	0.005	0.095	447	0.566	Y	8.046	42
At1g44575	Q9XF91	PSBS_ARATH Photosystem II 22 kDa protein, chloroplastic	0.43	0.24	0.011	0.27	438	0.555	Y	7.986	45
		**Stress response**									
At5g16710	Q8LE52	DHAR3_ARATH Glutathione S-transferase DHAR3, chloroplastic	0.24	0.66	0.042	0.361	293	0.54	Y	-1.085	40
At1g53280	Q9MAH3	DJ1B_ARATH Protein DJ-1 homolog B	1.86	Unique in Col-0	0.2	n.a.	525	0.578	Y	5.927	47
At4g08390	Q42592	APXS_ARATH L-ascorbate peroxidase S, chloroplastic/mitochondrial	1.41	0.38	0.528	0.027	911	0.543	Y	3.489	52
		**Redox regulation**									
At4g34120	Q9C5D0	CBSX2_ARATH CBS domain-containing protein CBSX2	0.52	0.3	0.085	0.017	262	0.562	Y	7.545	65
At1g76080	Q9SGS4	CDSP_ARATH Thioredoxin-like protein CDSP32	2.36	1.01	0.008	0.979	330	0.579	Y	4.894	43
		**Protein folding**									
At5g13410	Q9LYR5	FKB19_ARATH Peptidyl-prolyl cis-trans isomerase FKBP19, chloroplastic	n.e.v.	1.65	n.a.	0.039	469	0.551	Y	2.257	40
At2g43560	O22870	FK163_ARATH Peptidyl-prolyl cis-trans isomerase FKBP16-3, chloroplastic	0.69	0.6	0.168	0.019	916	0.565	Y	0.488	26
At5g50920	Q9FI56	CLPC1_ARATH Chaperone protein ClpC1, chloroplastic	0.8	0.91	0.052	0.553	256	0.543	Y	4.665	29
		**Cell wall**									
At1g11580	Q1JPL7	PME18_ARATH Pectinesterase/pectinesterase inhibitor 18	Unique in Col-0	0.59	0.037	0.045	469	0.563	Y	2.002	39
At5g64570	Q9FLG1	BXL4_ARATH Beta-D-xylosidase 4	4.5	0.58	0.053	0.549	486	0.501	Y	2.965	33
At3g29320	Q9LIB2	PHS1_ARATH Alpha-glucan phosphorylase 1	0.64	0.33	0.202	0.011	219	0.561	Y	7.918	48
		**Unknown**									
At4g13200	Q8LDV3	Y4320_ARATH Uncharacterized protein At4g13200, chloroplastic	n.d.	0.31	n.a.	0.024	1067	0.571	Y	4.826	66

At1g06690	Q94A68	Y1669_ARATH Uncharacterized oxidoreductase At1g06690, chloroplastic	0.11	0.71	0.02	0.564	491	0.553	Y	3.958	27
		**Chloroplast import**									
At5g16620	Q9FMD5	TIC40_ARATH Protein TIC 40, chloroplastic	0.61	0.28	0.152	0.036	312	0.583	Y	3.533	55
		**Metabolism**									
At1g24100	O48676	U74B1_ARATH UDP-glycosyltransferase 74B1	Unique in Col-0	0.49	n.a.	0.352	229	0.552	Y	4.079	50
At4g19710	O81852	AKH2_ARATH Bifunctional aspartokinase/homoserine dehydrogenase 2, chloroplastic	0.55	Unique in Col-0	0.241	n.a.	398	0.547	Y	5.866	46
At1g31860	O82768	HIS2_ARATH Histidine biosynthesis bifunctional protein hisIE, chloroplastic	Unique in *pldα1-1*	n.e.v.	n.a.	n.a.	748	0.561	Y	2.599	60
At4g39980	P29976	AROF_ARATH Phospho-2-dehydro-3-deoxyheptonate aldolase 1, chloroplastic	n.d.	Unique in *pldα1-2*	n.a.	n.a.	258	0.57	Y	5.364	42
At4g36810	P34802	GGPP1_ARATH Heterodimeric geranylgeranyl pyrophosphate synthase large subunit 1, chloroplastic	Unique in *pldα1-1*	n.e.v.	n.a.	n.a.	409	0.586	Y	5.577	36
At4g31990	P46248	AAT5_ARATH Aspartate aminotransferase, chloroplastic	1.97	2.07	0.051	0.031	430	0.564	Y	9.91	98
At5g64300	P47924	RIBA1_ARATH Bifunctional riboflavin biosynthesis protein RIBA 1, chloroplastic	n.d.	Unique in *pldα1-2*	n.a.	n.a.	741	0.533	Y	2.392	38
At3g01120	P55217	CGS1_ARATH Cystathionine gamma-synthase 1, chloroplastic	Unique in *pldα1-1*	Unique in *pldα1-2*	n.a.	n.a.	257	0.551	Y	7.395	44
At4g33510	Q00218	AROG_ARATH Phospho-2-dehydro-3-deoxyheptonate aldolase 2, chloroplastic	2.01	n.e.v.	0.026	n.a.	543	0.574	Y	5.392	56
At4g14210	Q07356	PDS_ARATH 15-cis-phytoene desaturase, chloroplastic/chromoplastic	n.d.	Unique in *pldα1-2*	n.a.	n.a.	265	0.557	Y	4.233	59
At3g02780	Q42553	IDI2_ARATH Isopentenyl-diphosphate Delta-isomerase II, chloroplastic	3.27	2.55	0.053	0.446	995	0.588	Y	11.096	60
At3g59760	Q43725	CYSKM_ARATH Cysteine synthase, mitochondrial	0.26	0.93	0.007	0.924	430	0.59	Y	7.219	63
At4g39970	Q680K2	GPPL1_ARATH Haloacid dehalogenase-like hydrolase domain-containing protein At4g39970	n.d.	Unique in *pldα1-2*	n.a.	n.a.	387	0.573	Y	2.968	41
At1g09795	Q8GSJ1	HIS1B_ARATH ATP phosphoribosyltransferase 2, chloroplastic	Unique in *pldα1-1*	n.e.v.	n.a.	n.a.	748	0.52	Y	5.465	40
At5g59420	Q93Y40	ORP3C_ARATH Oxysterol-binding protein-related protein 3C	n.e.v.	Unique in *pldα1-2*	n.a.	n.a.	453	0.547	Y	1.495	41
At2g31810	Q93YZ7	ILVH2_ARATH Acetolactate synthase small subunit 2, chloroplastic	Unique in *pldα1-1*	n.d.	n.a.	n.a.	302	0.58	Y	6.405	56
At4g38970	Q944G9	ALFP2_ARATH Fructose-bisphosphate aldolase 2, chloroplastic	0.78	0.87	0.013	0.188	238	0.589	Y	4.454	63
At5g11880	Q94A94	DCDA2_ARATH Diaminopimelate decarboxylase 2, chloroplastic	0.5	0.68	0.042	0.308	962	0.523	Y	2.411	63
At1g48610	Q94AD1	D1861_ARATH Putative DNA-binding protein At1g48610	n.d.	Unique in *pldα1-2*	n.a.	n.a.	174	0.563	Y	8.866	67
At4g35630	Q96255	SERB1_ARATH Phosphoserine aminotransferase 1, chloroplastic	0.8	0.41	0.662	0.003	442	0.592	Y	4.31	58
At5g19620	Q9C5J8	OEP80_ARATH Outer envelope protein 80, chloroplastic	Unique in Col-0	1.92	n.a.	0.102	464	0.555	Y	4.873	13
At5g23010	Q9FG67	MAM1_ARATH Methylthioalkylmalate synthase 1, chloroplastic	Unique in Col-0	0.66	n.a.	0.168	289	0.587	Y	3.262	71

At5g37510	Q9FGI6	NDUS1_ARATH NADH dehydrogenase [ubiquinone] iron-sulfur protein 1, mitochondrial	Unique in Col-0	0.72	n.a.	0.491	732	0.535	Y	1.493	93
At5g59290	Q9FIE8	UXS3_ARATH UDP-glucuronic acid decarboxylase 3	n.d.	Unique in *pldα1-2*	n.a.	n.a.	566	0.527	Y	4.032	75
At5g47840	Q9FIJ7	KAD2_ARATH Adenylate kinase 2, chloroplastic	Unique in *pldα1-1*	Unique in *pldα1-2*	n.a.	n.a.	223	0.567	Y	3.353	36
At5g55070	Q9FLQ4	ODO2A_ARATH Dihydrolipoyllysine-residue succinyltransferase component of 2-oxoglutarate dehydrogenase complex 1, mitochondrial	Unique in Col-0	0.39	n.a.	0.104	650	0.581	Y	1.907	38
At5g08530	Q9FNN5	NDUV1_ARATH NADH dehydrogenase [ubiquinone] flavoprotein 1, mitochondrial	0.38	0.44	0.001	0.296	281	0.525	Y	1.695	47
At1g32220	Q9FVR6	Y1222_ARATH Uncharacterized protein At1g32220, chloroplastic	1.25	3.05	0.774	0.031	489	0.576	Y	6.107	49
At5g10920	Q9LEU8	ARLY_ARATH Argininosuccinate lyase, chloroplastic	Unique in *pldα1-1*	n.d.	n.a.	n.a.	342	0.584	Y	5.062	65
At3g23940	Q9LIR4	ILVD_ARATH Dihydroxy-acid dehydratase, chloroplastic	Unique in Col-0	0.31	n.a.	0.04	671	0.505	Y	3.817	80
At1g05560	Q9LR44	U75B1_ARATH UDP-glycosyltransferase 75B1	0.74	0.27	0.57	0.026	256	0.597	Y	9.152	59
At3g19480	Q9LT69	SERA3_ARATH D-3-phosphoglycerate dehydrogenase 3, chloroplastic	0.53	0.65	0.459	0.001	223	0.537	Y	0.037	58
At3g61440	Q9S757	CYSC1_ARATH Bifunctional L-3-cyanoalanine synthase/cysteine synthase C1, mitochondrial	2.26	1.65	0.108	0.034	847	0.507	Y	12.729	12
At5g43780	Q9S7D8	APS4_ARATH ATP sulfurylase 4, chloroplastic	0.82	0.11	0.648	0.025	585	0.531	Y	2.006	54
At3g13750	Q9SCW1	BGAL1_ARATH Beta-galactosidase 1	n.e.v.	Unique in *pldα1-2*	n.a.	n.a.	507	0.559	Y	8.91	47
At5g51820	Q9SCY0	PGMP_ARATH Phosphoglucomutase, chloroplastic	0.55	0.59	0.035	0.092	633	0.524	Y	3.4	40
At4g37870	Q9T074	PCKA_ARATH Phosphoenolpyruvate carboxykinase (ATP)	Unique in *pldα1-1*	n.d.	n.a.	n.a.	238	0.581	Y	4.146	71
At4g23890	Q9T0A4	NDHS_ARATH NAD(P)H-quinone oxidoreductase subunit S, chloroplastic	1.09	Unique in Col-0	0.8	n.a.	517	0.569	Y	2.002	45
At4g01900	Q9ZST4	GLNB_ARATH Nitrogen regulatory protein P-II homolog	0.46	0.49	0.003	0.325	524	0.53	Y	5.945	32
At1g23740	Q9ZUC1	AOR_ARATH NADPH-dependent alkenal/one oxidoreductase, chloroplastic	0.49	0.98	0.022	0.895	457	0.506	Y	2.985	35
At2g43100	Q9ZW84	LEUD1_ARATH 3-isopropylmalate dehydratase small subunit 1	0.45	0.58	0.001	0.002	212	0.527	Y	2.351	17
At3g16000	Q9LW85	MFP1_ARATH MAR-binding filament-like protein 1	1.05	0.51	0.915	0.025	342	0.517	Y	-1.354	33
At1g31230	Q9SA18	AKH1_ARATH Bifunctional aspartokinase/homoserine dehydrogenase 1, chloroplastic	0.75	0.11	0.585	0.013	377	0.504	Y	8.208	31
At1g70730	Q9SGC1	PGMC2_ARATH Probable phosphoglucomutase, cytoplasmic 2	0.93	0.68	0.849	0.023	404	0.58	Y	-0.457	74
At1g62640	P49243	FABH_ARATH 3-oxoacyl-[acyl-carrier-protein] synthase III, chloroplastic	0.93	0.66	0.847	0.01	506	0.573	Y	5.708	49
At5g66120	Q8VYV7	DHQS_ARATH 3-dehydroquinate synthase, chloroplastic	0.62	0.41	0.397	0.036	490	0.546	Y	10.117	35
At4g26970	Q94A28	ACO2M_ARATH Aconitate hydratase 2, mitochondrial	3.16	3.27	0.17	0.054	372	0.588	Y	7.628	94
At3g57610	Q96529	PURA_ARATH Adenylosuccinate synthetase, chloroplastic	0.76	0.39	0.121	0.023	284	0.566	Y	4.26	45
